# Effects of research complexity and competition on the incidence and growth of coauthorship in biomedicine

**DOI:** 10.1371/journal.pone.0173444

**Published:** 2017-03-22

**Authors:** Jason Cory Brunson, Xiaoyan Wang, Reinhard C. Laubenbacher

**Affiliations:** 1 Center for Quantitative Medicine, UConn Health, Farmington, CT, United States of America; 2 Department of Family Medicine, UConn Health, Farmington, CT, United States of America; 3 Jackson Laboratory for Genomic Medicine, Farmington, CT, United States of America; Mälardalen University, SWEDEN

## Abstract

**Background:**

Investigations into the factors behind coauthorship growth in biomedical research have mostly focused on specific disciplines or journals, and have rarely controlled for factors in combination or considered changes in their effects over time. Observers often attribute the growth to the increasing complexity or competition (or both) of research practices, but few attempts have been made to parse the contributions of these two likely causes.

**Objectives:**

We aimed to assess the effects of complexity and competition on the incidence and growth of coauthorship, using a sample of the biomedical literature spanning multiple journals and disciplines.

**Methods:**

Article-level bibliographic data from PubMed were combined with publicly available bibliometric data from Web of Science and SCImago over the years 1999–2007. We selected four predictors of coauthorship were selected, two (study type, topical scope of the study) associated with complexity and two (financial support for the project, popularity of the publishing journal) associated with competition. A negative binomial regression model was used to estimate the effects of each predictor on coauthorship incidence and growth. A second, mixed-effect model included the journal as a random effect.

**Results:**

Coauthorship increased at about one author per article per decade. Clinical trials, supported research, and research of broader scope produced articles with more authors, while review articles credited fewer; and more popular journals published higher-authorship articles. Incidence and growth rates varied widely across journals and were themselves uncorrelated. Most effects remained statistically discernible after controlling for the publishing journal. The effects of complexity-associated factors held constant or diminished over time, while competition-related effects strengthened. These trends were similar in size but not discernible from subject-specific subdata.

**Conclusions:**

Coauthorship incidence rates are multifactorial and vary with factors associated with both complexity and competition. Coauthorship growth is likewise multifactorial and increasingly associated with research competition.

## Introduction

Coauthorship growth has been the subject of much research and commentary in biomedicine. Investigations by biomedical researchers into the patterns of coauthorship in their own literatures have accelerated, and, taken together, identified several factors that can account for much of the statistical variation in coauthorship rates. These investigations have figured primarily into two agendas: to gauge the extent to which an array of social and technological forces are responsible; and to determine how much of the growth in coauthorship is due to changing authorship norms or inappropriate authorship versus changes in the numerical extent of collaboration—that is, the number of people actively involved in a collaboration and deserving of authorship credit.

We build upon the first agenda: Its constituent studies are united by a sound demonstration of coauthorship growth itself, general consensus on several factors predictive of higher authorship, and common themes in the explanations and interpretations put forth. We suggest that the commonest explanations can be organized into two distinct accounts, and we examine bibliographic records through this lens in order to assess the relative support for these accounts.

### Background

We use “coauthorship incidence” as shorthand for the distribution of author counts across publications, and “coauthorship growth” for the secular trend in the center (mean, median, or mode) of this distribution. A steady rise in author counts has been documented in several specialties [1–10], in major journals [11, 12], and in biomedical research writ large [13–15]. Observers have proposed and tested a range of explanations, which roughly sort into the technical demands of frontier-expanding and boundary-spanning research and the incentives created by the selective allocation of scarce professional resources [16]. Borrowing from taxonomies by Katz and Martin [17], Barnett et al [18], and especially Pintér [19], we adopt the shorthands *complexity* and *competition* for these two accounts.

#### Complexity

The first account emphasizes the cognitive and time demands researchers face due to the accumulation of scientific knowledge and increasing sophistication of tools, and to the ability of researchers to meet these demands through specialization, division of labor, and reciprocity. This account derives much of its empirical support from studies that compare coauthorship incidence rates between disciplines, journals, or study types between which the researchers judge to be a complexity differential. For example, higher coauthor counts have been observed in studies using empirical versus theoretical designs [6], in multi-center versus single-center trials [20, 21], in multidisciplinary versus single-discipline studies [8], and (among clinical trials) in studies with larger sample sizes [22] and in general medicine versus surgery [23]. Coauthor counts have also been found to correlate with reference counts [24], which might be viewed as a proxy for the amount of background knowledge on which the study relies.

We focus on two factors extractable from our dataset: study type and topical scope. Several studies of coauthorship in biomedicine have classified articles by research protocol and measured the effect of the resulting scheme on coauthorship rates. For example, Borry et al [6], in an analysis of coauthorship growth in bioethics, included an indicator for whether each published study used an empirical design, and found that the rise of empirical research could account for most of the concurrent rise in coauthorship rates. In contrast, although Pintér [20] observed a rise in multi-center trials concurrent with rising coauthorship in the *European Journal of Pediatric Surgery*, when Khan et al [25] and Tilak et al [21] included an indicator for this study type in their analyses of obstetrics and gynecology and in top general medicine journals, respectively, they found little evidence that coauthorship growth could be accounted for by changes in the distribution of articles by study type. Topical scope, by which we mean the range of distinct topics a study bears upon, is not trivial to measure. Porter et al [26] provide a detailed discussion of measuring the related concept of interdisciplinarity using journal subject classifications. In biomedicine, several studies of coauthorship growth have compared patterns between disciplines [22, 23], but we are only aware of one that incorporates an article-level measure of scope: Barão et al [8] defined a collaborative article as one by authors from at least two specialties, though they did not examine the effect of this variable on author counts. We devised two measures of scope based on the controlled medical vocabulary used to tag articles in PubMed.

#### Competition

The second account emphasizes the pressures researchers face from evaluative criteria, which often rely on quantifiable accomplishments like grants and patents awarded, articles published, and the citation rates of publishing journals, that directly inform hiring, promotion, and tenure decisions and may indirectly affect social capital among colleagues. In partial support of the notion that demand for recognition incentivizes weaker criteria for authorship, several journals and repositories have placed restrictions on the number of authors to whom an article was credited or required authors to detail and affirm their contributions, which sometimes had discernible effects on author counts [3, 11, 12, 27–30].

For our analysis, we took advantage of funding tags and journal citation data; the role of editorial policies was assumed to be captured by journal-level effects. Clear evidence that grant-funded studies produced articles that credited more authors has been found in several biomedical disciplines [6, 8, 31]. Other studies have found that articles published in journals with higher impact factors tend to credit more authors [22, 32, 33]. Papatheodorou et al [22] read this as an indicator that more demanding research may be more attractive to prospective coauthors for its potential visibility, consistent with the interpretation of citation metrics as measures of journal popularity, rather than prestige [34].

### Objectives

This study addresses several limitations of existing work toward understanding authorship growth in biomedicine. First, previous analyses of multiple explanatory factors predominantly focused on coauthorship incidence [22, 25], while some compared coauthorship growth in different literatures using separate models [7, 35]. In order to measure the effects of our intervening variables on both incidence and growth, we included both main effects and date-of-publication interaction effects in a multiple regression model. Second, while the directions of the effects of many of these factors are known, their standardized magnitudes—indicators of their relative importance—have not been assessed. In order to directly compare their effects, we standardized each factor by its observed variation. Third, though coauthorship growth may vary widely from journal to journal within a single research domain [36], and from topic to topic within a single study type [22], the extent of this variation, in particular the representativeness of the journals and domains surveyed so far [14], is unknown. To improve representativeness, we drew our data without constraints on discipline or topic and systematized our journal selection process. To quantify the variation in incidence and growth across the literature, we incorporated the journal of publication into our statistical analysis as a grouping factor.

## Methods

### Data acquisition

We mined article-level bibliographic data directly from PubMed, including, for each article, the authors’ names; the date and journal of publication; the Medical Subject Headings (MeSH) assigned by the National Library of Medicine (NLM); and the publication type field, which included several types per typical article. We obtained journal-level bibliometric data from Thomson–Reuters Web of Science (WoS) [37] and SCImago [38], including the impact factor, the SCImago journal rank indicator, and the mean 2-year article citation count; and journal publication frequencies from the journal listings at PubMed.

We restricted our sample to research articles, using the “Study Type” field in PubMed to exclude editorials, letters, narratives, historical articles, addresses, comments, biographies, guidelines, directories, handouts, news items, and scientific integrity reviews. We further restricted to journals that published at least 30 research articles per year throughout our observation window and for which bibliometric data were available from both WoS and SCImago.

That observation window was 1999–2007. Two constraints limited us to these years: First, in contrast to many previous studies, we treat bibliometric indicators as longitudinal variables, in order to account for changes in journals’ citation rates. SCImago data prior to 1999 were not accessible in electronic form, so our observation window begins then. At the other end, the recent dramatic shift from print to electronic journal formats has seen the emergence of electronic-only journals, the transition of existing print journals to hybrid or electronic form, and the cessation of many print-only journals. This phenomenon may have impacted coauthorship rates and, we think, deserves consideration as a factor. However, our restriction to continuously-publishing journals had the effect of excluding the majority of electronic journals. To avoid possible confounding, we opted to exclude electronic journals from the analysis. We ended our observation window with the onset, in 2007, of a decline in research articles published in print journals. Another factor worth noting is that, from 1996 to 1999, PubMed capped listed author counts at 25. This overlaps with our observation window in only one year, and no more than 55 articles—less than 0.1%—in any year from 2000 to 2007 credited more than 25 authors. We therefore ignore the inconsistency.

### Variable selection

**Fig 1:**
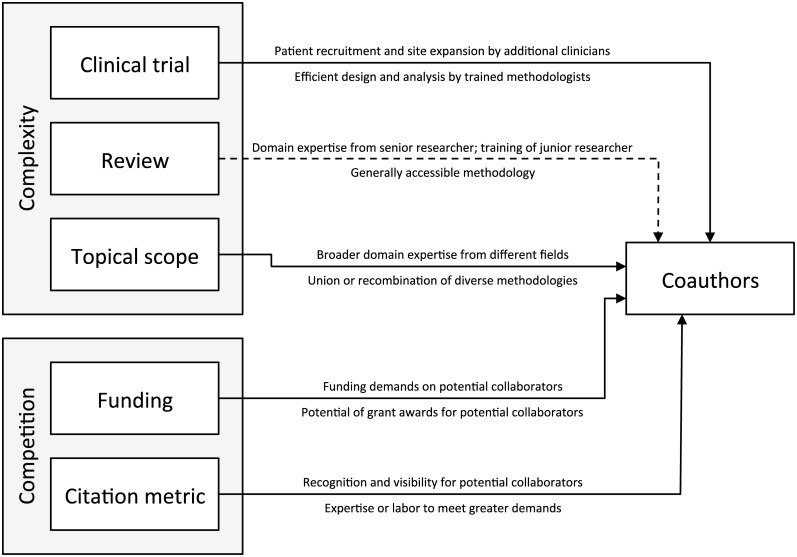
Conceptual model of predictors and response. Solid lines indicate positive effects; the dashed line indicates a negative effect. The binning of predictors into “complexity” and “competition” is synthesized from previous biomedical studies and commentaries.

Our response variable was the number *NC*_*i*_ of authors, *in addition to the first*, credited by the *i*^th^ article. This made *NC*_*i*_ a count variable, and a measure of coauthorship we call the coauthor count. We coded the journal of publication *j*[*i*] as its (print) ISSN and the date of publication *DP*_*i*_ as the number of years since January 1999 in intervals of $\frac{1}{12}$ (monthly), the finest resolution shared by all articles in the dataset. Fig 1 summarizes how we conceive of the causal relationships between the other predictors, described in more detail below, and the number of authors credited by an article. We considered multiple measures of some variables, for instance the Thomson–Reuters impact factor and the SCImago journal rank indicator as measures of journal popularity. Our choices for the main analysis were made to minimize the pairwise correlations between the input variables. In a sensitivity analysis, we reproduced the analysis for each combination of measures and noted any differences in the results.

#### Study type

Our analysis would be strengthened by discriminable indicators of study type—that is, indicators that more evenly partition our sample. This is for two reasons: They reflect more substantive methodological categories with straightforward interpretations; and, as binary rather than categorical variables, they produce more discernible effects. With this in mind, we adopted the indicator variable *CT*_*i*_, for whether article *i* was classified as any type of clinical trial, as were 5.6% of the articles in our dataset (a larger proportion than for other study types). Clinical trials often rely on a time-consuming process of subject recruitment and follow-through and involve sophisticated statistical machinery to determine design parameters for the trial and detect signals in the data; consistent with the complexity account, we expect the articles reporting their findings to credit more authors than average. We also included an indicator variable *RV*_*i*_ for whether article *i* was classified as a review (9%), as some other recent analyses have done [33, 35]. Literature reviews involve less elaborate data collection and limited, if any, technical analysis (meta-analysis being an exception), and are often conducted by senior experts in a field working alone or with mentees, and therefore credit fewer authors on average.

#### Research support

PubMed includes several Research Support tags in the Publication Type field, specific to funding obtained from the NIH (intramural and extramural), the Public Health Service, other government sources, and non-government sources, which include governments other than the United States. In keeping with the discriminability principle, we defined the indicator variable *RS*_*i*_ by whether article *i* received research support from any source, which characterized 59% of the articles in our sample. One problem with this measure is that it tags articles written by NIH researchers receiving intramural NIH funding. Since the other tags are not partitioned this way, however, we opted not to specifically exclude these. As an alternate measure of funding, we considered whether article *i* acknowledged at least one grant, according as the Grant Number field was not empty (16%).

#### Topical scope

The scope of a research project, for instance in terms of the disciplines it draws from or contributes to or of the topics it concerns, is notoriously difficult to measure. When a bibliographic database employs a journal- or article-level subject classification scheme, it may be used to construct a measure of multi- or inter-disciplinarity, for instance by counting the codes assigned to an article [39], by characterizing the distribution of codes assigned to all of a researcher’s articles [40], or by weighing the co-assignment of codes to journals by their rarity [26, 41]. We attempted something similar using the controlled MeSH vocabulary, which NLM assigns to articles indexed by PubMed based on their subject content for retrieval purposes. In addition to broad disciplinary categories, headings include biological species, clinical procedures, statistical techniques, laboratory equipment, and other more varied topical indicators. We therefore interpret the number of headings assigned to an article as a measure of its topical scope.

There are many qualifications to be made here: MeSH was designed to standardize terminology; headings may be more plentiful in different disciplines of similar scope due to differences in jargon or the need to interface with other disciplines, and the human staff who assign them must use their subjective judgment. Because the vocabulary is hierarchical (unlike WoK subject classifications, for example), higher-level headings often appear above more granular subheadings. Additionally, some headings may be tagged as major, i.e. focal to the article. The latter two issues prompted us to refine the simple heading count in two ways: We took *MT*_*i*_ to be the number of major headings assigned to article *i*, which we used in the main analysis. As an alternate measure, we calculated the number of distinct top-level headings by excluding all subheadings.

#### Journal popularity

To the extent that higher-impact journals publish articles that credit more authors but are otherwise similar to others, we interpret this as evidence that the competition for professional recognition incentivizes coauthorship. We conceive of a core research team that initiates a project, then may seek additional contributors with desired expertise at different stages of the research process. (A similar conceptualization has been used to explain the distribution of coauthors credited by physics research articles [42].) If the current authors expect the study to be published in a higher-IF journal, then they are likely to include more auxiliary work, in anticipation of (or possibly in response to) greater demands from reviewers, and seek additional contributors to conduct it. They will also find it easier to attract these contributors, who will themselves be more incentivized to seek authorship credit (e.g., rather than an acknowledgment).

There are other mechanisms by which the coauthor count and the publishing journal’s IF may be related. For example, articles by more authors tend to receive more citations, contributing to the IF one to two years later; however, the already-weak correlation between individual articles’ citation rates and their publishing journals’ impact factors declined previously to and during our study interval [43], suggesting that this causal pathway is insignificant. Other factors, such as the composition of the research team, are also known to predict the IF of the publishing journal [44]. Furthermore, as discussed in the Background section, citation metrics like IF measure a journal’s popularity rather than its prestige. This calls into question some past interpretations of the effect of the publishing journal’s IF on the number of authors credited by an article [22], including our own. Since these interpretations are based on authors’ attitudes toward the IF, it remains plausible that prestige, rather than popularity, provides the incentive. A popularity incentive could be interpreted through either of our lenses—complexity (the authors expect a wider audience to find their work useful) or competition (the authors seek greater recognition for their work). There is also conceptual disagreement, in the analysis of other disciplines, over whether increased competition incentivizes collaborative or solo authorship [45, 46]; by and large, however, previous researchers have shared our interpretation of the *IF*–*NC* relationship as a positive sign of competition.

We denote by *IF*_*j*_ the impact factor of journal *j*, and we write *j*[*i*] for the journal that published article *i*. We also considered two alternate measures of journal influence: the SCImago journal rank indicator and the raw 2-year citation rate (obtained from SCImago).

#### Publishing journal

Other journal-level properties are known or thought to affect coauthorship rates among the articles a journal publishes, including editorial policies and discretion, the domain, field, and/or specialty it serves, and the habits and norms of its community of contributors. As an extreme case, the *Canadian Journal of Cardiology* jumped from an average 2.9 authors per article in 2002 to 4.3 in 2003, in contrast to steady incidence rates before and after. More generally, different coauthorship patterns have been observed between domains, fields, and specialties, some of which we reproduce in our supplemental analyses. The publishing journal is one of several ways these factors might be incorporated, but it is also of particular interest itself: Most studies of coauthorship growth in biomedicine have drawn from small samples of journals, which has raised concerns that their results may not be representative of the whole. Including the publishing journal as a random effect allows us to directly address this concern, without burdening the model with hundreds of journal-specific indicator variables or diluting the sample by performing the analysis for only one journal at a time.

### Regression analyses

Regression models of coauthorship historically belong to three families: classical linear regression, using author count [18, 21, 22, 47] or average author count by discipline or journal [32]; logistic regression, using an indicator that the number of authors exceeds some cutoff [8, 25]; and Poisson regression, using coauthor count [18, 45, 46]. In order to take advantage of within-journal variation, we opted not to aggregate our data into within-journal averages; in order to detect the changing shape of the author count distribution, we opted to treat it as a count variable. This suggests a Poisson regression model.

However, Beaver [48] observed that the cross-sectional distribution of author counts in a range of scientific literatures has grown overdispersed, relative to the earlier more nearly Poisson shape, so that it is better modeled by a negative binomial (NB) distribution. We corroborated this observation for the biomedical literature: The cross-sectional distributions for different years were substantially overdispersed, relative to the equivalent Poisson distribution [49], while the NB model fit the cross-sectional data reasonably well [50] (see S1 Text). While we didn’t find the NB to be a perfect fit, we did find it much more suitable than the Poisson, or than other distributions for count data used in regression [51]. We therefore adopted a NB regression framework, using the canonical log link function.

We log-transformed impact factors to *LIF*_*j*[*i*]_ in order to linearize their relationship with $\log({\bar{NC}}_{jd})$, taking mean author counts ${\bar{NC}}_{jd}$ over journal–years (all articles *i* for which *j*[*i*] = *j* and *DP*_*i*_ = *d*). Since many articles were assigned no major MeSH terms, we used the transformation *LMT*_*i*_ = log(*MT*_*i*_ + 1) to linearize the relationship between topical scope and $\log({\bar{NC}}_{m})$, this time taking mean author counts conditionally on *MT*_*i*_ = *m*. We centered these input variables *LMT* at its median value, corresponding to *MT* = 4, and *LIF* at its mean, corresponding to *IF* = 2.271. Publication date is an interval, not a ratio, variable, so we did not conceive of it as occupying any “natural” range, as was necessary for the predictors we sought to directly compare. We therefore did not standardize *DP*, but we did center it at January 2003, so that incidence and growth rates are calculated relative to that month.

In a preliminary analysis, we tracked the effect estimates of the article-level factors (other than *DP*) in cross-sectional models for each year from 1999 to 2007. The main analysis assumes linear growth (or decline) in each effect *β*_*X*_ over time, and this allowed us to check this assumption.

For the main analysis, we fit two NB multiple regression models. Both included interaction terms of *DP*_*i*_ with each of the other input variables. These interaction effects are interpretable from two perspectives: With respect to the effect *β*_*DP*_ of the publication date, *β*_*DP*×*X*_ can be viewed as the effect of *X* on coauthorship growth; with respect to the effect *β*_*X*_ of the factor *X*, *β*_*DP*×*X*_ can be viewed as the growth rate of the effect of *X* on coauthorship incidence.

The fixed-effects model has the form

$$\begin{array}{ccc} \text{log}\left(E\left[NC_{i}\right]\right) & = & \beta_{1}+\beta_{DP}DP_{i}\\ & + & \left(\beta_{RS}+\beta_{DP\times RS}DP_{i}\right)RS_{i}\\ & + & \left(\beta_{CT}+\beta_{DP\times CT}DP_{i}\right)CT_{i}\\ & + & \left(\beta_{RV}+\beta_{DP\times RV}DP_{i}\right)RV_{i}\\ & + & \left(\beta_{LMT}+\beta_{DP\times LMT}DP_{i}\right)LMT_{i}\\ & + & \left(\beta_{LIF}+\beta_{DP\times LIF}DP_{i}\right)LIF_{j\left[i\right]} \end{array}$$

with the effects coming in pairs (intercept and *DP*, then main and *DP*-interaction effects of the other predictors). The mixed model

$$\begin{array}{ccc} \text{log}\left(E\left[NC_{i}\right]\right) & = & \beta_{1}+\beta_{DP}DP_{i}\\ & + & \left(\beta_{RS}+\beta_{DP\times RS}DP_{i}\right)RS_{i}\\ & + & \left(\beta_{CT}+\beta_{DP\times CT}DP_{i}\right)CT_{i}\\ & + & \left(\beta_{RV}+\beta_{DP\times RV}DP_{i}\right)RV_{i}\\ & + & \left(\beta_{LMT}+\beta_{DP\times LMT}DP_{i}\right)LMT_{i}\\ & + & \left(\beta_{LIF}+\beta_{DP\times LIF}DP_{i}\right)LIF_{j\left[i\right]}\\ & + & \left(b_{1j\left[i\right]}+b_{DP,j\left[i\right]}DP_{i}\right) \end{array}$$

additionally includes, at the level of each journal *j*, random intercept and *DP* effects

$$\begin{array}{ccc} B_{1j} & ∼ & N\left(0,\sigma_{1}{}^{2}\right)\\ B_{DP,j} & ∼ & N\left(0,\sigma_{DP}{}^{2}\right)\text{,} \end{array}$$

from which *b*_1*j*_ and *b*_*DP*,*j*_ are taken to have been drawn [52–54]. The random effects are added to the intercept *β*_1_ and *DP*-slope *β*_*DP*_ to produce the main effects specific to each journal. The procedure returns conditional modes ${\hat{b}}_{1j}$ and ${\hat{b}}_{DP,j}$, which predict the individual journals’ deviations in coauthorship incidence and growth rate from that of the aggregated literature (adjusting for other factors). We first checked the distributions of the ${\hat{b}}_{1j}$ and the ${\hat{b}}_{DP,j}$ for deviations from Gaussianity. The model includes the additional parameters ${\sigma_{1}}^{2}$ and ${\sigma_{DP}}^{2}$, which may be estimated as the sample standard deviations of the conditional modes [52]. We derived no expectations from the literature about the relationship between coauthorship incidence and growth across journals. In the model, therefore, we allowed for correlation between the random effects. This meant estimating not only the journal effect variances ${\hat{\sigma }}_{1}^{2}$ and ${\hat{\sigma }}_{DP}^{2}$ but the full covariance matrix [52].

For the main effects of the numerical variables *X* = *LMT*, *LIF*, we plot standardized effect estimates ${\hat{\beta }}_{X}/2s_{X}$, obtained as the estimated effects scaled by twice the sample standard deviations, on the principle that this scaled effect indicates the difference in responses between articles having “low” and “high” values of these variables, analogously to the values 0 and 1 of the indicator variables [55]. We took the standard deviation of log–impact factor over journal–years, though this value differed little (< 5%) from that taken over articles. For interaction effects with publication date, we again scaled down by 2*s*_*X*_. (Since every interaction term comes from pairing another predictor *X* with *DP*, it makes as much sense to directly compare the ${\hat{\beta }}_{DP\times X}/2s_{X}$ as the ${\hat{\beta }}_{X}/2s_{X}$.) We took the values $2{\hat{\sigma }}_{1}$ and $2{\hat{\sigma }}_{DP}$ as rough indicators of the difference in coauthorship incidence and growth between articles published in journals at the low and high ends of each distribution, to be (cautiously) compared to the standardized effect estimates.

Before fitting the models, we partitioned the full dataset into a training set (50%) and a testing set. We ensured that the training set included at least 15 articles in each journal each year. We fit each model to the training set, measured its goodness of fit by the Akaike information criterion [56], and measured its predictive accuracy by its mean squared error (MSE) on the testing set. We did not perform hierarchical model selection because the sheer size of the dataset led almost any null hypothesis test to reject at a minuscule false positive rate. However, we did use a method of Burnham and Anderson [56] to compare the importance, in information-theoretic terms, of each component of the final model (see S1 Text).

We performed all calculations and produced all images in the statistical programming language R [57], using the data.table package [58] to process data and the lme4 package [52] to fit generalized linear mixed models. Code to reproduce the analysis is available on GitHub (https://github.com/corybrunson/coauthor), and the preprocessed data is available on Zenodo (https://zenodo.org/deposit/345934).

## Results

### Summary of the data

The final dataset described 589,681 articles in 283 journals, ranging in average publication volume (for articles meeting our inclusion criteria) from 47.3 articles per year (*Artificial Intelligence in Medicine*) to 1229.8 articles per year (*Transplantation Proceedings*). Corroborating many previous studies, we observe an acceleration in the number of articles published each year. We further point out that, since the journal subset was fixed, this acceleration is not an artifact of journal proliferation or turnover. However, especially in the second half of our observation window, the growth (and acceleration) in publications was limited to those that received financial support. Indeed, the number of unsupported review and clinical trial–based articles *decreased* from 2004 to 2007 (see S1 Text).

**Fig 2:**
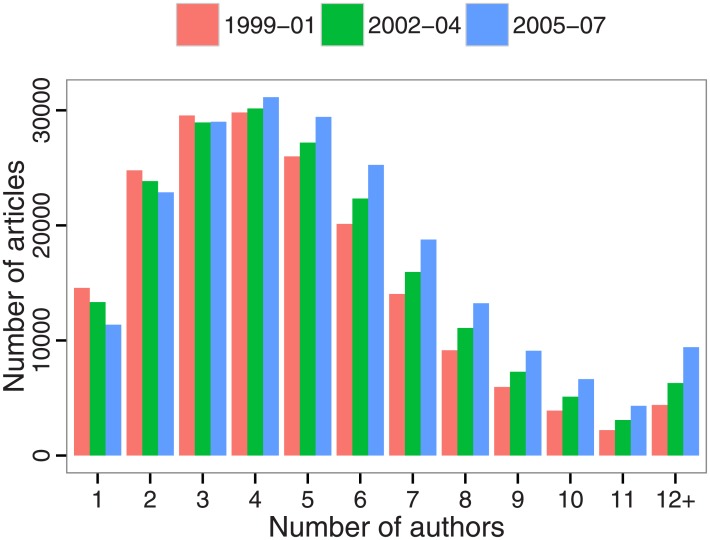
Distribution of author counts, stratified by 3-year interval. The bars of a single color depict the distribution for a single interval, and articles with 12 or more authors are binned together.

Both the signature shape and the gradual shift of the author count distribution are discernible in Fig 2. One- and 2-author articles are not just on the decline; their declines are outpaced by the rise of articles by 5, 6, 7, or 8 authors, as well as by growth in the cumulative number of articles by 12 authors or more. A proportional histogram stratified in the same way, in which the sum of the heights of the bars for each interval total 100%, reveals that the changing shape of the distribution “pivots” about 4- and 5-author papers, which comprised about 15% and 13% of articles over each interval, respectively.

Table 1 summarizes the sample across odd-numbered years. Coauthorship evolved in tandem with several other trends, including a research literature coded to increasingly many simultaneous topics, a growing reliance on external funding, and a rise in citation rates. Except for the decline in the proportion of clinical trial reports, the trends were consistent with coauthorship growth, based on previous research. In particular, on average, journals saw increasing citation rates and articles broadened in disciplinary scope. However, while journals and articles grew more homogeneous (as measured by variance) in these terms, they grew more heterogeneous in terms of coauthorship. The decoupling of these variables calls into question their explanatory relationship.

**Table 1:** Biannual distributions of variables across all articles in our sample.

	1999		2001		2003		2005		2007	
Clinical trial										
*CT* = 0	55802	0.939	59374	0.947	60623	0.940	64906	0.938	66779	0.948
*CT* = 1	3650	0.061	3355	0.053	3867	0.060	4272	0.062	3691	0.052
Review										
*RV* = 0	54023	0.909	56917	0.907	58301	0.904	62918	0.910	64309	0.913
*RV* = 1	5429	0.091	5812	0.093	6189	0.096	6260	0.090	6161	0.087
Support										
*RS* = 0	25263	0.425	26652	0.425	26805	0.416	27547	0.398	26614	0.378
*RS* = 1	34189	0.575	36077	0.575	37685	0.584	41631	0.602	43856	0.622
Major MeSH										
*NMT* ≤ 2	11947	0.201	11580	0.185	10239	0.159	10601	0.153	9809	0.139
2 < *NMT* ≤ 5	37566	0.632	38697	0.617	40749	0.632	43717	0.632	45058	0.639
5 < *NMT*	9939	0.167	12452	0.199	13502	0.209	14860	0.215	15603	0.221
Mean (SD)	3.9 (1.7)		4.1 (1.8)		4.2 (1.8)		4.2 (1.7)		4.3 (1.6)	
Impact factor										
*IF* ≤ 1	8427	0.142	7719	0.123	5626	0.087	5255	0.076	3742	0.053
1 < *IF* ≤ 3	32712	0.550	33908	0.541	33907	0.526	33370	0.482	36479	0.518
3 < *IF*	18313	0.308	21102	0.336	24957	0.387	30553	0.442	30249	0.429
Mean (SD)	3.0 (3.3)		3.2 (3.3)		3.4 (3.4)		3.5 (3.2)		3.4 (2.9)	
Coauthor count										
*NC* ≤ 2	13042	0.219	12781	0.204	12162	0.189	11414	0.165	11111	0.158
2 < *NC* ≤ 5	28099	0.473	28599	0.456	28631	0.444	29858	0.432	29540	0.419
5 < *NC*	18311	0.308	21349	0.340	23697	0.367	27906	0.403	29819	0.423
Mean (SD)	4.6 (2.8)		4.8 (2.9)		5.1 (3.1)		5.4 (3.4)		5.5 (3.6)	

In rows labeled by logical criteria, the integers indicate numbers of articles, while the fractions indicate proportions of the total. In rows labeled “Mean (SD)”, the values are the means and standard deviations.

**Fig 3:**
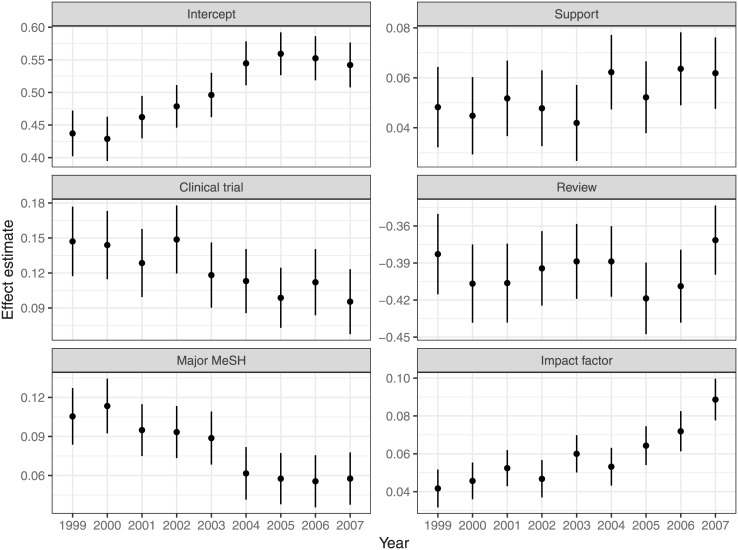
Effect estimates in cross-sectional fixed-effects models of coauthorship for each year in our observation window, with 99% confidence intervals. The estimates have not been standardized; except for the intercepts, they may be compared to the estimates in Table 2.

Fig 3 tracks the cross-sectional estimates for these factors over our observation window; in each case, the estimates vary only within a factor of 2. The cross-sectional intercepts, which govern the baseline coauthorship rates in the models when the predictors are held fixed at their centers (or zero, for the binary predictors), increased steadily over most of our observation window, suggesting a secular trend not explained by the predictors; this was confirmed by the main models. We also perceive definite trends in three of the article-level predictors: research suppport (upward), major MeSH count (downward), and journal impact factor (upward), supporting the inclusion of *DP*-interaction effects in the full models.

### Effect estimates

The main effect and *DP*-interaction effect estimates for each model constitute Table 2. Estimates ${\hat{\beta }}_{X}$ and ${\hat{\beta }}_{DP\times X}$ from separate “single-confounder” models

$$\log\left(E\left[NC_{i}\right]\right)=\beta_{1}+\beta_{DP}DP_{i}+\left(\beta_{X}+\beta_{DP\times X}DP_{i}\right)X_{i}\text{,}$$

taking *X* to be each input variable besides *DP*, are included for comparison. The estimates involving *MT* and *IF* have been de-standardized, so that they may be interpreted as estimates of parameters governing exponential relationships and are not directly comparable. In each case the direction of the effect is consistent with expectations. Most interaction effects are smaller than their respective main effects by an order of magnitude or more, indicating that fundamental shifts in their effects (e.g. doubling or vanishing) take decades, but nevertheless most are statistically discernible. Below we report these effects in percentage terms; a more technical discussion is included in S1 Text.

**Table 2:** Main and interaction effect estimates.

	Single confounder		Fixed effects		Mixed effects	
Predictor	Main	*DP*×	Main	*DP*×	Main	*DP*×
Intercept (2003)	–	–	1.349 (.003)	.018 (.001)	1.241 (.016)	.023 (.002)
Clinical trial	.312 (.008)	−.014 (.003)	.251 (.008)	−.013 (.003)	.186 (.007)	−.012 (.003)
Review	−.856 (.008)	.004 (.003)	−.793 (.008)	.001 (.003)	−.728 (.008)	.000 (.003)
log(Major MeSH + 1)	.296 (.006)	−.016 (.002)	.169 (.005)	−.016 (.002)	.129 (.005)	−.005 (.002)
Support	.210 (.004)	.005 (.001)	.104 (.004)	.004 (.002)	.146 (.004)	.006 (.001)
log(Impact factor)	.136 (.003)	.010 (.001)	.112 (.003)	.009 (.001)	.032 (.012)	.006 (.002)

Main effect and date-of-publication (*DP*) interaction effect estimates, with standard errors, for each predictor coupled with DP and in both models of the main analysis. The top row shows each model’s intercept estimate ${\hat{\beta }}_{1}$ and *DP* main effect estimate ${\hat{\beta }}_{DP}$. We measured *DP* in years, so ${\hat{\beta }}_{DP}$ is an estimated annual coauthor growth rate.

#### Date of publication

We estimated aggregate coauthor growth rates at 1.8% ± 0.1% (fixed-effects) or 2.3% ± 0.2% (mixed-effects) per year, which accumulate to 20% or 26% growth per decade. Whereas the median coauthor count throughout our observation window was 4, this translates to a secular trend of about one additional author per decade, consistent with previous estimates [14, 22]. These estimates were attenuated, though for the most part slightly, by other input variables. Growth rate estimates were not dramatically different in simpler models (not shown): The simple model with *DP* as the sole predictor returns an estimate of 2.74% ± 0.07%, and the multiple regression model having the same input variables but no interaction terms returns 2.15% ± 0.07%.

#### Study type

The most dramatic difference we observed in coauthorship rates was between review and non-review articles. Relative to other articles, review articles credited 45% as many coauthors, or 55% fewer coauthors (52% fewer after accounting for journal effects). Articles reporting on clinical trials tended to credit 29% more coauthors (20% after accounting for journal effects). While the review article effect remained stable, our predicted clinical trial effect shrank at a rate of about 1.3% per year.

#### Research support

We estimated a smaller positive effect of research support than of the study type indicators. An article that received support tended to have 11% more coauthors than one that did not (16% after accounting for journal effects), and this gap was slowly growing.

#### Topical scope

We estimated *β*_*LMT*_ at 16.9% ± 0.5%, or 12.9% ± 0.5% after accounting for journal effects, which indicates that an article assigned 2 major terms (the low end) tends to have only 92% as many coauthors as (or 8% fewer coauthors than) one assigned 4, and 13% fewer than one assigned 6 (the high end). These effects may appear slight or substantial, depending on other factors.

#### Journal popularity

We estimated *β*_*LIF*_ at 10.9% ± 0.2%, which indicates that an article in a journal with low IF 1.085 has 8% fewer coauthors than one published in a journal with typical IF 2.271, and 15% fewer than one published in a journal with high IF 4.755.

#### Publishing journal

In the mixed-effects model, we incorporated the publishing journal, as a grouping factor with associated random intercept and *DP* effects. We estimated that the journal-level intercepts, or 2003 base authorship rates, varied with standard deviation ${\hat{\sigma }}_{1}=.259$, which is to say that we predict otherwise similar articles published in journals at the low end of this distribution (*b*_1*j*_ = −.259) to have only about 77% as many coauthors as, or 33% fewer coauthors than, those published in an average journal and those at the “high” end (*b*_1*j*_ = .259) to have 130% as many, or 30% more. Relative to the fixed effects, then, the publishing journal had a large influence on coauthorship rates. We estimated ${\hat{\sigma }}_{DP}=.013$, meaning that the growth rates at the “low” and “high” ends of the distribution were about 1.3% per year lower and higher, respectively, than average. The distributions of conditional modes are depicted in quantile plots in S1 Text. The distribution of ${\hat{b}}_{1j}\text{s}$ is symmetric but leptokurtic, though the deviation is mild. The distribution of ${\hat{b}}_{DP,j}\text{s}$ is more nearly Gaussian, except for rapid coauthorship growth in five outlying journals.

The expected percentage differences in coauthor counts between articles taking low versus high values of each predictor are summarized in Table 3. The main effects of major MeSH count, clinical trial design, and especially journal impact factor are noticeably reduced in the mixed-effects model. This confounding is consistent with the reasonable expectations that specific journals tend to publish articles with similar disciplinary breadth, that certain journals specialize in publishing clinical trial reports, and that journals’ impact factors are stable over time. However, we note that the year-to-year variation in *LMT* within journals (84–88%) is much greater than that between journals (12–16%), indicating that journals are not meaningfully tiered with respect to our measure of topical scope and calling into question a disciplinary (or otherwise journal-correlated) interpretation of *MT*.

**Table 3:** Percentage changes in expected coauthor counts.

	Reference values		Model	
Predictor	“Low”	“High”	Fixed effects	Mixed effects
Clinical trial	No	Yes	28.5%	20.4%
Review	No	Yes	−54.8%	−51.7%
log(Major MeSH + 1)	2	6	15.4%	11.5%
Support	No	Yes	11.0%	15.7%
log(Impact factor)	1.085	4.755	18.0%	4.8%
Journal	−1×SD	1×SD	–	67.9%

Percentage differences in expected coauthor counts due to increasing each predictor from its “low” to its “high” value, as described in the Regression analyses section. For the publishing journal, the value is the percentage difference in expected coauthor count due to changing from a journal *j* with *b*_1*j*_ = −.259 to one with *b*_1*j*_ = .259.

**Fig 4:**
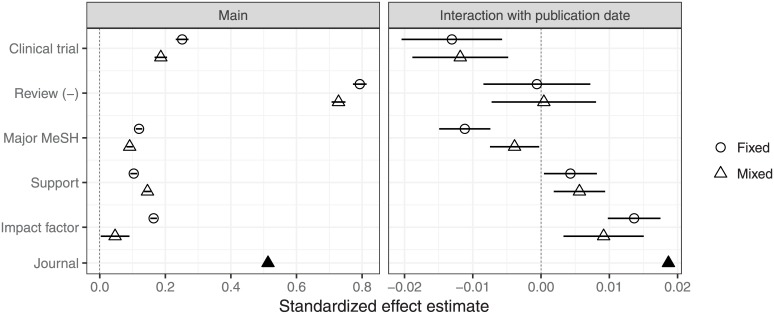
Direct comparisons of standardized main effect and date-of-publication (*DP*) interaction effect estimates, with 99% confidence intervals. The effects of the binary variables are the same as in Table 2, except that the effects of review (*β*_*RV*_ and *β*_*DP*×*RV*_) are negated to facilitate comparisons of the absolute values of the main effects. The numerical variables were scaled by twice their sample standard deviations before fitting the models, so their effects plotted here are unitless. Higher interaction effects indicate that an input variable is more positively associated with coauthor growth. The values plotted for “Journal” are twice the estimated standard deviations of the journal-level random intercept (2003) and *DP* interaction (growth rate) effects, 2*σ*_1_ and 2*σ*_*DP*_. They are not interpretable in the same way—in particular, they are unsigned and have no associated standard errors—but convey a rough sense of the relative predictive importance of the journal of publication.

The interaction effects confirm the pattern suggested by the cross-sectional estimates in Fig 3: With the exception of ${\hat{\beta }}_{DP\times RV}$, which is not discernibly nonzero, the *DP*-interaction effects associated with the complexity account (${\hat{\beta }}_{DP\times CT}$ and ${\hat{\beta }}_{DP\times MT}$) are negative, while those associated with the competition account (${\hat{\beta }}_{DP\times RS}$ and ${\hat{\beta }}_{DP\times IF}$) are positive. As suggested by the ranges of their cross-sectional estimates, the right frame of Fig 4 shows that the predictive strengths of the *DP*-interaction effects (excluding ${\hat{\beta }}_{DP\times RV}$) were similar. Notably, more coauthorship growth may be attributable to within-journal patterns (${\hat{\sigma }}_{DP}$) than to any of the article-level predictors.

We found the journal-level incidence and growth rates to be uncorrelated (*r* = −0.04), indicating that, controlling for other factors, the most rapid coauthorship growth occurred in journals across the spectrum in terms of coauthorship incidence. This suggests that journals are not diverging or polarizing with respect to their coauthorship rates. We further discerned at best a weak relationship (*r* < 0.3) between either the incidence or the growth in coauthorship and journal publication volume. (Correlations with *IF* would not be meaningful since this was a predictor in the mixed model.)

The left frame of Fig 4 plots the standardized effect estimates ${\hat{\beta }}_{X}/2s_{X}$ for both models (excluding those of 1 and *DP*), together with $2{\hat{\sigma }}_{1}$, on a common scale for comparison. By far the strongest predictor of author count is being a review article (the negatives of its estimates plotted in order to make visual comparison easier). In the mixed-effects model, the publishing journal is almost as strong a predictor; though the review article effect accounts for a great deal of variation that would otherwise figure into the publishing journal, since many review articles are published by review-oriented journals. The next-strongest, though much weaker, predictor is being based on a clinical trial; the remaining estimates, in both models, are of similar size.

The right frame of Fig 4 similarly plots the estimates ${\hat{\beta }}_{DP\times X}/2s_{DP\times X}$, together with $2{\hat{\sigma }}_{DP}$. The coauthor growth rate for review articles is not discernibly different from the aggregate growth rate. Those reporting on clinical trials had a lower growth rate, by about the same margin as those with financial support had a higher growth rate. Having been assigned more major MeSH terms was associated with similarly reduced growth, but, as with the main effect, this estimate shrunk substantially after accounting for journal effects. The effect of impact factor was similar, in the opposite direction. The publishing journal itself had the most pronounced effect, but accounting for it did not qualitatively change the results.

### Subject-specific analyses

The main analysis conflates patterns in fields of study throughout the domain of biomedicine. To test for variation across fields, we took advantage of the Subject Classifications (SCs) used by WoS, as proxy indicators of fields of study, to classify journals (see S1 Text for a table of frequencies). We fit the fixed- and mixed-effects models of the main analysis to 32 subsets of data, obtained by restricting the publishing journals to those assigned each of 32 distinct SCs. (We used only SCs assigned to at least six journals in our dataset. A single journal may receive multiple SCs and therefore figure into multiple of these subset analyses.) The results for some SCs run counter to our main results, confirming that differences in research practice and culture between fields play a role that is not captured by our primary variables of interest, but the broad patterns are consistent with them. The results are summarized in S1 and S2 Figs. While both the main effects and the *DP*-interaction effects were similar in size, in almost all cases the *DP*-interaction effects were statistically indiscernible. This shows that our results could not have been obtained from datasets like those used in past analyses, which were usually drawn from single disciplines and at most a handful of journals.

### Sensitivity analyses

We performed sensitivity analyses to assess the importance of several analysis decisions we made, and to address some additional concerns from the Introduction section. The changes made to the analysis presented here are as follows: (i) extending the interval to 1997, the earliest year for which WoS data were available; (ii) removing articles written by corporate authors, as an imperfect proxy for consortium authors, from the dataset; (iii) substituting alternative measures of several variables, described in the Variable selection section; (iv) relaxing the restriction to journals indexed by WoS (i.e. for which IFs were available) and introducing a binary variable for whether each article’s publishing journal was indexed; (v) treating IF as a journal- as well as an article-level predictor by including a linear term for LIF on the journal-level random effects in the mixed model; (vi) assuming a lognormal, rather than a NB, model of the coauthor count distribution [59]. We report full results in S1 Text. By and large, the results reported above are robust to these choices, with the exception that several alternative approaches produced subtle changes in the estimated effects of the complexity-related predictors: Lengthening the time interval to 1997 rendered the *DP*-interaction effect of *LMT* statistically indiscernible (in the mixed model), and both ${\hat{\beta }}_{DP\times CT}$ and ${\hat{\beta }}_{DP\times LMT}$ indiscernible. Meanwhile, vastly expanding the sample to journals not indexed by WoS resulted in a discernibly positive interaction effect ${\hat{\beta }}_{DP\times RV}$, so that all three complexity-related factors weakened over time (since ${\hat{\beta }}_{RV}$ was negative).

## Discussion

We designed our approach to address some common limitations of previous studies of coauthorship growth in biomedicine. Specifically, we aimed to draw a more representative sample of the biomedical literature, to estimate the effects of several known factors on both the incidence and the growth of coauthorship, to quantify the variation across journals, and to compare the influence or importance of the factors.

By including hundreds of journals in our analysis, we were able to describe the distribution of coauthorship rates—both incidence and growth—among journals serving dozense of disciplines and across a spectrum of popularities, rather than compare rates between selected journals in separate disciplines or among top-tier journals in a single discipline. We observed these distributions, controlling for study type, impact factor, and some other factors, to be symmetric and unimodal, though not necessarily Gaussian. These rates reflected a log-transformed author count variable; their exp-transformed distributions, particularly that of the incidence effects, were right-skewed, consistent with the core–periphery hypothesis, in which an elite subset of journals publishes characteristically high-authorship papers while the majority of remaining journals publish relatively few. Comparisons of the standard deviations of the journal-level random effects and the standardized effects of article-level predictors suggests that the journal of publication may be a stronger predictor than most article-level factors, though this may be largely a function of the study’s (hence the journal’s) discipline. These observations partially address a concern of Levsky et al [14]. However, we corroborate the weaker hypothesis that coauthorship tracks journal popularity, in terms of citation rates, and in fact we show that this association has been strengthening. Beaver [60] has cautioned against drawing inferences about scientific practice in general from patterns observed in a select few “core” journals or even those indexed by bibliometric databases, and this caution remains relevant.

Our observation that the distribution of author counts did not just increase, but also grew more dispersed, was consistent with other studies [19, 32, 35]. The contrasting reduced variance of the numerical predictors—major MeSH term count, as a proxy for topical scope, and journal impact factor, as a measure of popularity—indicate that these variables would do little to explain rising authorship. This was confirmed in the regression analyses, which estimated similar aggregate growth rates with and without accounting for the input variables—that is, a secular trend remains to be adequately explained. These growth rate estimates were also consistent with those obtained in other studies [22].

### Complexity and competition

Our results corroborated the previously identified effects of several factors: In addition to more topic-spanning articles and those published in more popular journals, articles that reported on clinical trials and that received financial support tended to credit more authors. By considering the contributions of these factors to the growth rate as well as the incidence rate of coauthorship, we found that the variables associated with complexity, when they had discernible effects on authorship growth, slowed it; whereas the variables associated with competition accelerated it. This suggests that, though both complexity and competition contribute to coauthorship incidence, competition is driving coauthorship growth; though we emphasize that our analysis was not designed to support causal inference. This pattern did not change after accounting for journal-level effects, or through any of several sensitivity analyses. The main results suggested that the effects of complexity-related factors on coauthorship rates are decreasing, but multiple sensitivity analyses indicated that this observation is not robust.

**Fig 5:**
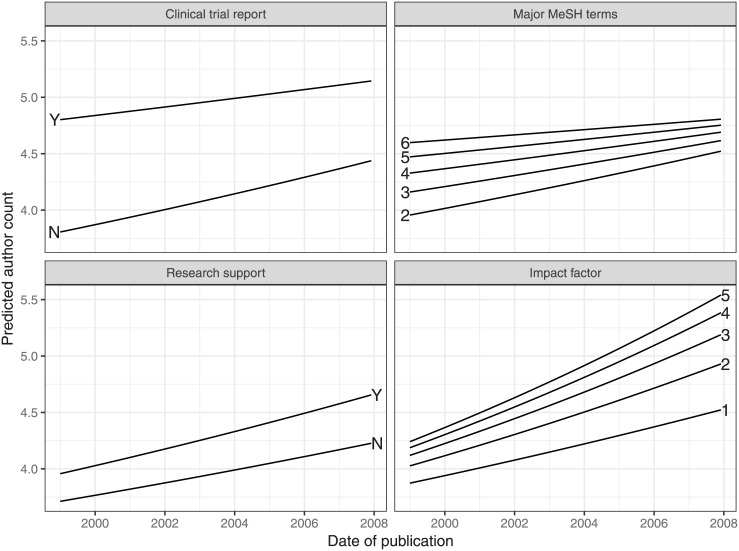
Changing effects of four input variables on predicted author count over time. Each variable ranges over several benchmark values (*CT* = 0, 1; *IF* = 1, 2, 3, 4, 5; *RS* = 0, 1; *NMT* = 2, 3, 4, 5, 6) while the others are held constant at their means. Coauthor growth has been slower in clinical trial reports and in more topic-spanning articles, but quicker in financially-supported studies and in articles published in higher-impact journals.

The trends can be observed in the prediction curves of Fig 5: As time progresses, our fitted model predicts less dispersed coauthor counts by study type and topical scope, but more dispersed coauthor counts by financial support and journal popularity. That is, differential coauthorship is becoming more aligned with the hierarchies of funding and visibility and less aligned with those of methodology and scope.

Extrapolating these growth effects backward in time suggests that the incidence effects of competition-related factors would have been near zero at some point during the 1980s. This is inconsistent with the direct evidence from previous studies that both financial support and journal impact metrics were associated with higher coauthorship incidence before this time. Instead, we infer from the rapid increase in these incidence effects that they began having an outsized effect only relatively recently. This rapid increase, it must also be remembered, was only discernible due to the immensity of our dataset. An analysis of publications during the subsequent 8 years would help determine whether the increase was localized to the nineties and aughts or continued apace.

### Limitations

This study has several limitations that could not be addressed by sensitivity analysis. Our sample of journals and our period of observation were limited by the availability of citation metrics, which may have captured transient patterns. For example, this interval coincided with the five-year acceleration and subsequent deceleration of NIH funding, which had disorienting effects on aspects of research culture well beyond the grant application process [61, 62]. In the broader context of paradigmatic shifts, our results should be seen as cross-sectional rather than longitudinal; that is, we observed trends in the determinants of coauthorship growth characteristic of a specific period of time, which are likely to have been different at other moments over the course of biomedical research. (A comparison to Clarke [63] is illustrative.) This is especially important in light of our exclusion of the most recent 9 years of biomedical research, an interval characterized not only by a shift in publishing practices but also by rapidly changing technological, health, and political forces. Finally, some of our choices of variable measures—hence also our interpretations of their effect estimates—must be considered provisional: Previous interpretations of IF as an incentive for or against bringing more authors onto a project are inconsistent, and our measures of topical scope are original and have not been validated.

### Conclusion

Coauthorship growth in biomedical research has been investigated widely and frequently, and a coherent set of broad accounts has emerged through which most studies of the phenomenon are interpreted, in particular the increasing technical sophistication of the research process and attendant specialization of individual researchers (complexity) and the growing importance of scarce resource allocation to researchers and of recognition and visibility to researchers competing for it (competition). However, while evidence implicates each of these cultural trends in propelling coauthorship growth, no coherent picture has emerged of how they may reinforce or frustrate each other, or even of whether their have changed over time.

This study sketches a larger picture: We confirm the effects of study type, funding, and journal popularity, previously limited to specific disciplines, journals, or topics. We show that rates of both coauthorship incidence and coauthorship growth are distributed unimodally across journals, with a pronounced right skew in the case of incidence, and that the publishing journal is a better predictor than many properties of the articles themselves. Finally, we provide strong evidence that, while both complexity and competition play a role in producing higher author counts on published research articles, the role of complexity held steady or declined in the aughts, while the role of competition increased markedly. Competition is widely favored, and widely misgiven, as an explanation in commentaries on coauthorship growth in biomedicine. To our knowledge, this study provides the first bibliographic evidence for its ascendance as the primary driver.

## Supporting information

S1 Text Discussion of several auxiliary analyses supporting assumptions, simplifications, and other claims in the main text. This document contains several tables and figures with accompanying expository text.
(PDF)

S1 Fig Comparison of standardized main and *DP* interaction effect estimates from the fixed-effects model, with 99% confidence intervals, for each subset of at least six journals assigned a common Subject Classification (SC) in WoS. The SCs are listed first in order of the number of journals in our dataset to which they were assigned, then alphabetically. See S1 Text for the number of journals in each SC. See S1 Text for the number of journals in each SC.
(PDF)

S2 Fig Comparison of standardized main and *DP* interaction effect estimates from the mixed model, with 99% confidence intervals, for each subset of at least five journals assigned a common Subject Classification (SC) in WoS. The SCs are listed first in order of the number of journals in our dataset to which they were assigned, then alphabetically. See S1 Text for the number of journals in each SC.
(PDF)
