# Supplementary material for: Effects of research complexity and competition on the incidence and growth of coauthorship in biomedicine
Source: PLoS One. 2017 Mar 22;12(3):e0173444. doi: 10.1371/journal.pone.0173444 (PMC5362051; doi:10.1371/journal.pone.0173444)
Supplement: S1 Text — This document contains several tables and figures with accompanying expository text. (PDF) [file pone.0173444.s001.pdf]

Supplement to
"Coauthorship incidence and growth in biomedical research"

Jason Cory Brunson, Xiaoyan Wang, and Reinhard C. Laubenbacher

March 9, 2017

Methods

Outlying journals Figure 1 depicts, for each journal each year, its mean coauthor count versus its impact factor. Note the exceptional infrequency of coauthorship in *Scientific American*, a general science journal targeted to a popular audience. We omitted *SciAm* from the main analysis because it is unrepresentative of the peer-targeted scientific literature.

We highlight two (disjoint) subsets of the remaining journals: those published by the Nature Publishing Group (NPG), which were disproportionately cited, and those that publish mostly (> 50% over the observation period) review articles, including several in the Elsevier *Trends* series. The scatterplot illustrates the importance of including a review article indicator variable, and suggests that brand indicators may be warranted regressors, for instance because they capture editorial policies that apply to multiple journals.

Frequency of categories in analysis dataset A reasonable concern is that the disciplinary scope of PubMed extends well beyond disciplines properly classified with "biomedicine". We address this concern by tabulating, in Tables 1 and 2, the frequencies of each Subject Classification (SC) used in Thomson–Reuters Web of Knowledge (WoK) across the journals included in the main analysis. Most of the classifications are clearly appropriate for an inclusive analysis of biomedical literature. Several unexpected SCs occurred with low frequency, but on inspection the journals to which they had been assigned were judged appropriate to the scope of biomedicine. For example, 2 journals each were assigned the SCs "Chemistry, Physical" and "Computer Science, Interdisciplinary & Applications", which turned out to be *Biophysical Chemistry* and the *Journal of the American Society for Mass Spectrometry*, and *Medical & Biological Engineering & Computing* and *Computer Methods and Programs in Biomedicine*, respectively.

A related concern is how representative the journals indexed in PubMed, Web of Science, and SCImago (and, hence, the articles they publish) are of the entire biomedical literature. In each case, indexing decisions are based on quality assessments of journals, which likely correlate with citation metrics and plausibly with other variables of interest. We are unaware of research that directly addresses this question, but we partially address it later in this supplement with a sensitivity analysis that includes a binary indicator of inclusion in each bibliometric database (WoS and SCImago).

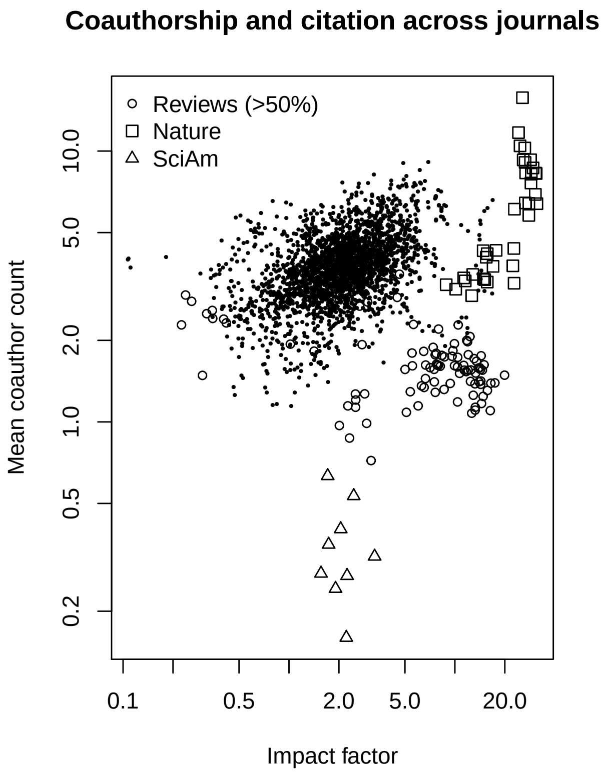

Figure 1: Mean coauthor count versus impact factor across journal–years. (Note the log–log scale.)

Category	No.
Biochemistry & Molecular Biology	35
Pharmacology & Pharmacy	31
Neurosciences	27
Cell Biology	15
Immunology	15
Oncology	14
Clinical Neurology	13
Endocrinology & Metabolism	13
Veterinary Sciences	12
Psychiatry	11
Radiology, Nuclear Medicine & Medical Imaging	11
Biotechnology & Applied Microbiology	10
Public, Environmental & Occupational Health	10
Hematology	9
Microbiology	9
Surgery	9
Engineering, Biomedical	8
Medicine, General & Internal	8
Medicine, Research & Experimental	8
Toxicology	8
Biochemical Research Methods	7
Biophysics	7
Dentistry, Oral Surgery & Medicine	7
Obstetrics & Gynecology	7
Pediatrics	7
Biology	6
Genetics & Heredity	6
Infectious Diseases	6
Ophthalmology	6
Orthopedics	6
Otorhinolaryngology	6
Sport Sciences	6
Geriatrics & Gerontology	5
Pathology	5
Peripheral Vascular Disease	5
Virology	5
Behavioral Sciences	4
Cardiac & Cardiovascular Systems	4
Chemistry, Analytical	4
Chemistry, Medicinal	4
Critical Care Medicine	4
Environmental Sciences	4
Medical Informatics	4
Parasitology	4
Physiology	4
Reproductive Biology	4
Respiratory System	4
Transplantation	4

Table 1: WoK Subject Classification frequencies (greater than 3) for journals included in this study.

Category	No.
Chemistry, Multidisciplinary	3
Dermatology	3
Food Science & Technology	3
Health Care Sciences & Services	3
Mathematical & Computational Biology	3
Nuclear Science & Technology	3
Nutrition & Dietetics	3
Plant Sciences	3
Tropical Medicine	3
Anatomy & Morphology	2
Chemistry, Inorganic & Nuclear	2
Chemistry, Physical	2
Computer Science, Interdisciplinary Applications	2
Medical Laboratory Technology	2
Spectroscopy	2
Substance Abuse	2
Urology & Nephrology	2
Acoustics	1
Agriculture, Dairy & Animal Science	1
Audiology & Speech-language Pathology	1
Chemistry, Applied	1
Chemistry, Organic	1
Computer Science, Artificial Intelligence	1
Computer Science, Information Systems	1
Computer Science, Theory & Methods	1
Developmental Biology	1
Ecology	1
Education, Scientific Disciplines	1
Electrochemistry	1
Emergency Medicine	1
Engineering, Civil	1
Engineering, Environmental	1
Entomology	1
Evolutionary Biology	1
Fisheries	1
Gastroenterology & Hepatology	1
Integrative & Complementary Medicine	1
Materials Science, Biomaterials	1
Microscopy	1
Nanoscience & Nanotechnology	1
Neuroimaging	1
Physics, Atomic, Molecular & Chemical	1
Psychology	1
Rehabilitation	1
Zoology	1

Table 2: WoK Subject Classification frequencies (at most 3) for journals included in this study.

Exploratory analysis We note, in disclosure, that we originally omitted RV_i as a predictor. Preliminary results revealed asymmetries in the distributions of journal-level random effects, which hampered model fitting and interpretation. These were found to be due largely to review-oriented journals, including the Elsevier *Trends* series. Once controlled for RV_i, the distributions of journal-level effects became much more symmetric.

Figure 2 reveals no dramatic changes in the relative proportions of articles over the 9-year period by research support or type of study. As many other studies have observed, sheer article number accelerated; however, in the latter part of our observation window growth has been limited to supported research within each study type. However, as one reviewer suggested, this may be due in part to supporting agencies increasingly ensuring, or ensuring with increasing success, that they are credited.

Figure 3 shows the evolving distribution of log-transformed impact factors across journals, and Figure 4 shows that of untransformed major MeSH term counts. The distributions share two trends: increasing center (whether median or mean) and decreasing spread (inter-quartile range or standard deviation). The pattern remains when the journal–years in the first distribution are weighted by article volume. That is, while journals are increasing their influence and articles are addressing more topics, they are also becoming more homogeneous in these respects. This is in marked contrast to the evolution of author counts depicted in Figure 1 in the main text.

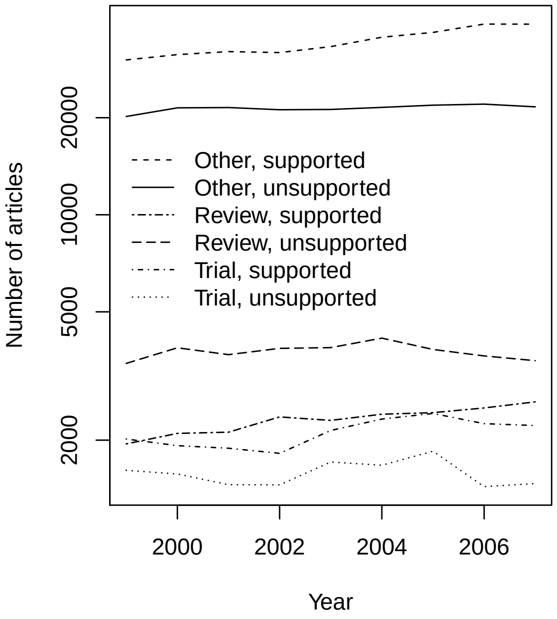

Figure 2: Article count each year, stratified by research support and two publication types. Note the logarithmic vertical scale.

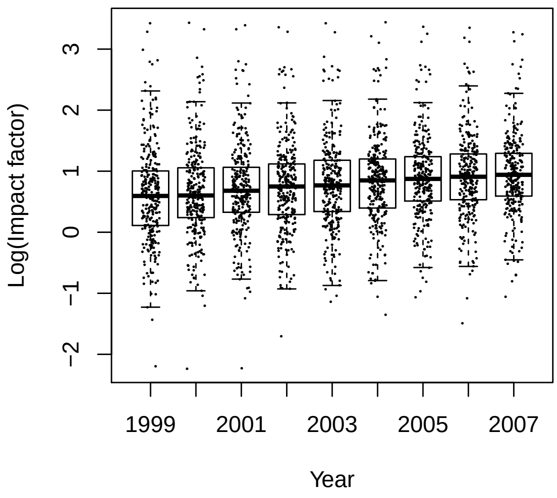

Figure 3: Distribution of log–impact factor over time. Each dot indicates one journal's log–impact factor in one year. A boxplot for each year is overlaid; the boxes contain the interquartile range (IQR) while the whiskers extend to 1.5 times the IQR from the boxes in each direction.

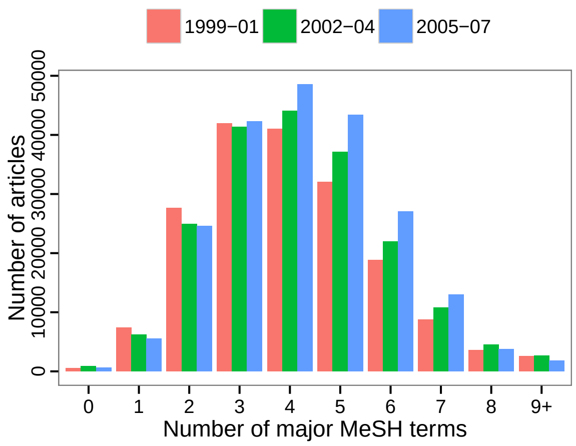

Figure 4: Distribution of major MeSH term count, stratified by 3-year interval. The bars of a single color depict the distribution for a single interval, and articles with 12 or more authors are binned together.

Distribution of coauthor counts Figures 5 and 6 visually test whether the coauthor counts NC_i in the starting and ending years of our observation window are distributed according to a Poisson or a negative binomial (NB) model, respectively. The Poissonness plots show a poor fit, due to overdispersion in the tails as well as at $k = 0$—that is, in addition to many more several-author articles than a Poisson process would predict, we (still) find many more single-author articles than the same model would predict. The NB model provides a much better fit, though noise in the tail obscures this as measured by each simple linear fit.

Figure 7 visualizes the overdispersion of the coauthor count distribution over time, relative to what would be expected under a Poisson model by comparing the variation to its value in a Poisson distribution (the mean). Not only is the distribution consistently overdispersed, with $s_{NC}/\overline{NC} > 2$ since 1999, but it has increased, such that $s_{NC}/\overline{NC} > 3$ since 2005. This trend can be traced back several decades, to a moment when coauthor counts were not far removed from a Poisson approximation (not shown), but the incompleteness of PubMed data cautions against drawing conclusions about biomedical research practice from this observation.

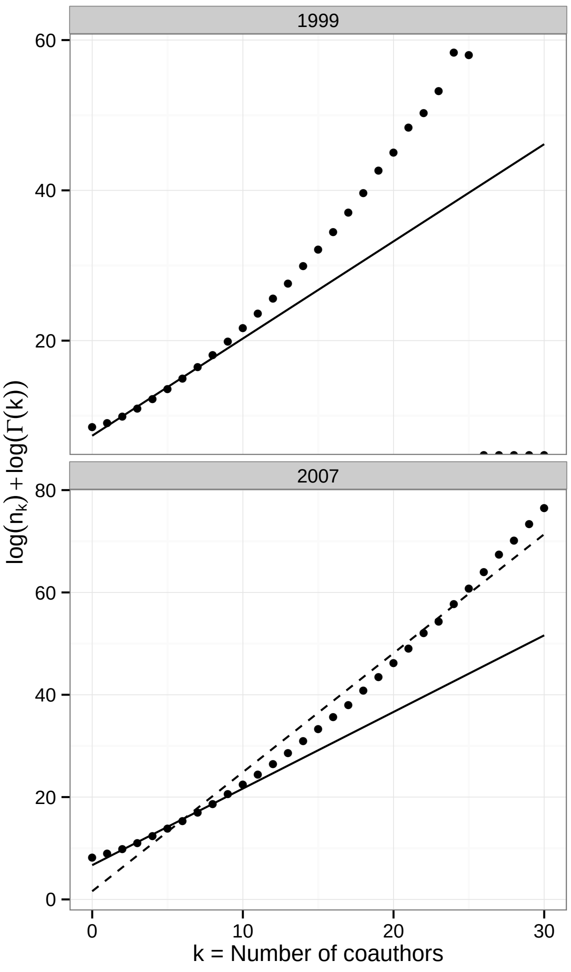

Figure 5: Poissonness plots for the coauthor count distribution in the starting and ending years of our observation window.

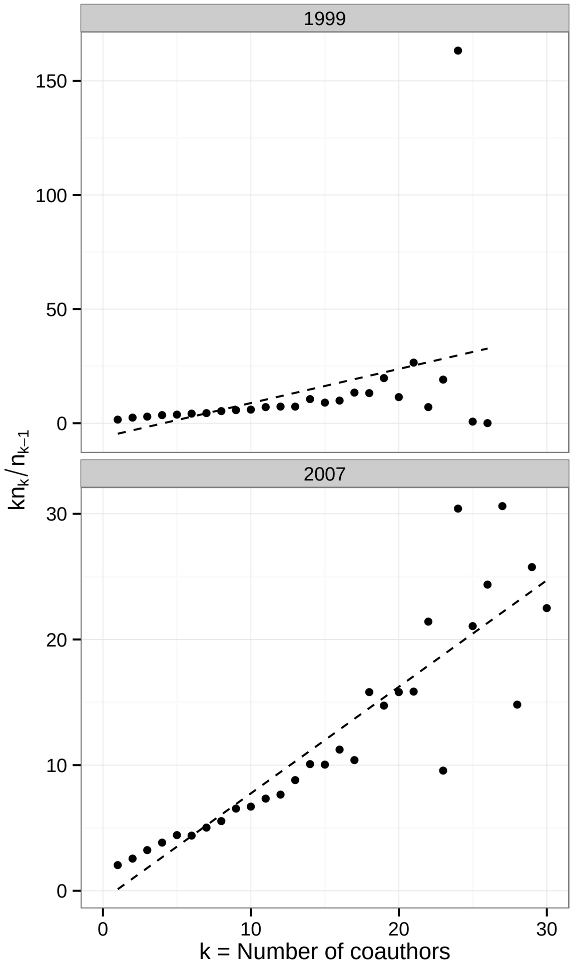

Figure 6: Ord NB plots for the coauthor count distribution in the starting and ending years of our observation window.

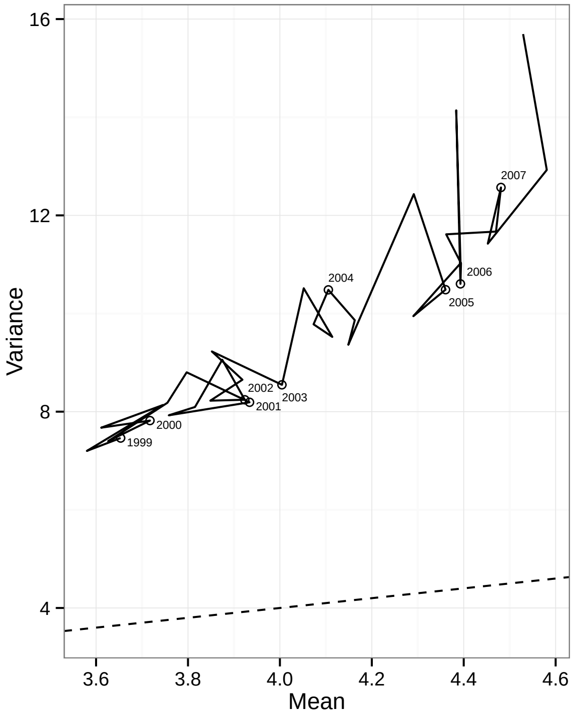

Figure 7: Sample variance s_{NC} versus sample mean $\overline{NC}$ of the coauthor count distribution in each quarter of the observation period. The ordered pair for the first quarter (Jan–Mar) of each year is circled and labeled by the year. The dashed line depicts the relationship $s_{NC} = \overline{NC}$ which would be expected if the cross-sectional distribution were Poisson.

Results

Interpretation of estimates The main text, in the Results section, communicated our results in terms of percentage differences in expected coauthor counts between articles at the high versus low values of a single predictor, holding other predictors fixed. (It does not matter at which values the other predictors are held when reporting percentage differences.) Here we illustrate how the model—specifically, the mixed-effects model—and the raw effect estimates translate into these percentages. Analogous translations can be calculated in the same way from the estimates obtained for the fixed-effects model.

First, the estimate $\hat{\beta}_{DP} = 0.023$ figures into the model as

$$\log(E[NC_i]) \approx 0.023 \times DP_i + C,$$

where C is the sum of the remaining terms; exponentiating both sides yields

$$E[NC_i] = \exp(0.023 \times DP_i + C) = Ke^{0.023 \times DP_i},$$

where $K = \exp(C)$, i.e. a continuous annual growth at a rate of 2.3% per year.

The binary predictors have simpler interpretations. For example, the estimate $\hat{\beta}_{RV} = -0.728$ yields $E[NC_i] = \exp(-0.728 \times RV_i + C) = K \times 0.48^{RV_i}$, which means that being tagged as a review article corresponds to crediting $(K \times 0.48^1)(K \times 0.48^0) = 48\%$ as many coauthors, or 52% fewer.

The numerical predictors require more care. The log-plus-one–transformed LMT term produces the relationship $E[NC_i] = \exp(\beta_{LMT} \log(MT + 1) + C) = K(MT+1)^{\beta_{LMT}}$, so that our estimate $\hat{\beta}_{LMT} = 0.129$ implies that, relative to an article assigned 4 terms (the median), an article assigned 6 terms is expected to credit $7^{0.129}/5^{0.125} = 104\%$ as many coauthors, or 4% more, while an article assigned 2 is expected to credit $3^{0.129}/5^{0.129} = 94\%$ as many, or 6% fewer. The log-transformed LIF term similarly produces $E[NC_i] = K \times IF^{\beta_{LIF}}$, so that the estimate $\hat{\beta}_{LIF} = 0.032$ implies that, relative to an article published in a middling-impact journal ($IF = 2.271$), an article published in a high-impact journal ($IF = 4.755$) is expected to credit 2.4% more authors, while an article published in a low-impact journal ($IF = 1.085$) is expected to credit 2.3% fewer.

Importance of terms In addition to the modeling framework, we compared the importance of each component (term) of the final model—the five linear predictors, their associated interaction effects, and the two journal-level random effects—in terms of information retention from the raw output values to the model predictions. We fit the model defined by each subset S_i of terms to the same data, this time consisting of one third of the full dataset (to reduce computation time) and calculated AIC_i for each. We then calculated the Akaike weights w_i, defined as the values $\exp(-\Delta_i/2)$, normalized to sum to 1, where the smallest AIC of any model equals $AIC_{\min}$ and $\Delta_i = AIC_i - AIC_{\min}$. For each model component x, the sum of the weights for models that incorporate x, which is strictly bounded below by 0 and above by 1, measures the importance of x. These measures are presented in Table 3. Note that, while most values are indistinguishable from 1, in reality orders of magnitude separate their respective differences from 1.

Component	Importance	Component	Importance
β_{CT}	1.00000	$\beta_{DP \times CT}$	0.99019
β_{RV}	1.00000	$\beta_{DP \times RV}$	0.27588
β_{LMT}	1.00000	$\beta_{DP \times LMT}$	0.74787
β_{RS}	1.00000	$\beta_{DP \times RS}$	1.00000
β_{LIF}	0.99999	$\beta_{DP \times LIF}$	0.54076
σ_1	1.00000	σ_{DP}	1.00000

Table 3: Information-theoretic importance of each component in the full model, fitted to a $\frac{1}{3}$-size subset of the dataset.

The full model had the third-lowest AIC, at 880,182.6; the two lowest were the model with $\beta_{DP \times RV} DP_i RV_i$ omitted (880,180.7) and the model with both $\beta_{DP \times RV} DP_i RV_i$ and $\beta_{DP \times LIF} DP_i LIF_{j[i]}$ omitted (880,181.1). Our non-null interpretations of the interaction effect $\beta_{DP \times LIF}$ should be taken with additional caution; we make none of $\beta_{DP \times RV}$.

Journal-level random effects Figure 8 assesses the Gaussianity of (the conditional mode predictions of) the journal-specific rates of coauthorship occurrence (top) and growth (bottom). The distribution of occurrence rates is thinner-tailed than Gaussian, as indicated by the downward (respectively, upward) concavity of the plotted pairs to the left (respectively, the right) of the center. That of the growth rates is visually indistinguishable from Gaussian, except that the most rapidly-growing coauthorship rates are higher than expected. This might suggest the need to identify and include conscious changes in editorial policies on author credit in future analyses. Extremal conditional modes for both random effects are presented in Table 4 with the journals they correspond to. Note the lack of correlation between the two random effects in this sample, consistent with the low correlation reported in the main text.

	Intercept	Pub. date
Acad Med	-0.51	0.03
Genetics	-0.22	0.03
Nat Genet	0.86	0.02
J Med Chem	0.69	-0.02
Curr Biol	-0.42	-0.02
Presse Med	0.03	0.03
Math Biosci	-0.82	0.00
Eur J Hum Genet	0.58	-0.02
J Morphol	-0.77	-0.00
Can Vet J	-0.74	0.01
J Biotechnol	0.05	-0.02
Biosystems	-0.74	0.00
Brain Res Bull	-0.17	0.02
J Neurol Sci	0.21	-0.02
Bone Marrow Transplant	0.69	-0.00
J Nucl Med	0.34	-0.02
Seizure	-0.13	0.02
Int J Mol Med	0.30	0.02
Proc Biol Sci	-0.52	0.01
AIDS	0.43	-0.01

Table 4: Twenty journals with the largest random effects. The journals are ranked by the sum of squares of their standardized effects.

While the conditional modes are roughly symmetrically distributed, they are not necessarily the values of interest: Since the conditional modes act as effects in a log-transformed model of expected coauthor counts, the distribution of actual average author counts across journals, controlling for the other factors used in the mixed model, is better estimated by their exponentials. Figure 9 reproduces Figure 8 using the exponentiated conditional modes $\exp(\hat{b}_{1j})$ and $\exp(\hat{b}_{DP,j})$, from which the difference can be discerned. While the distribution of date-of-publication journal-level effects remains approximately symmetric, that of the random intercepts, which are linearly related to the journal-specific average numbers of authors per article, is skewed right. In particular, the approximately linear quantile–quantile relationship abruptly steepens around the 60th percentile and again around the 90th percentile. While these sharp changes may be artifacts of our data and analysis, the skewness itself is consistent with a tiered model of scientific output, in which higher levels of collaboration are concentrated within a limited network that publishes in a select subset of journals, while lower—though by no means zero—levels of collaboration are typical of the remaining output. It must be emphasized, however, that the stratification appeared *after* controlling for journal impact factor; the mechanism behind it may be distinct from citation-measured influence.

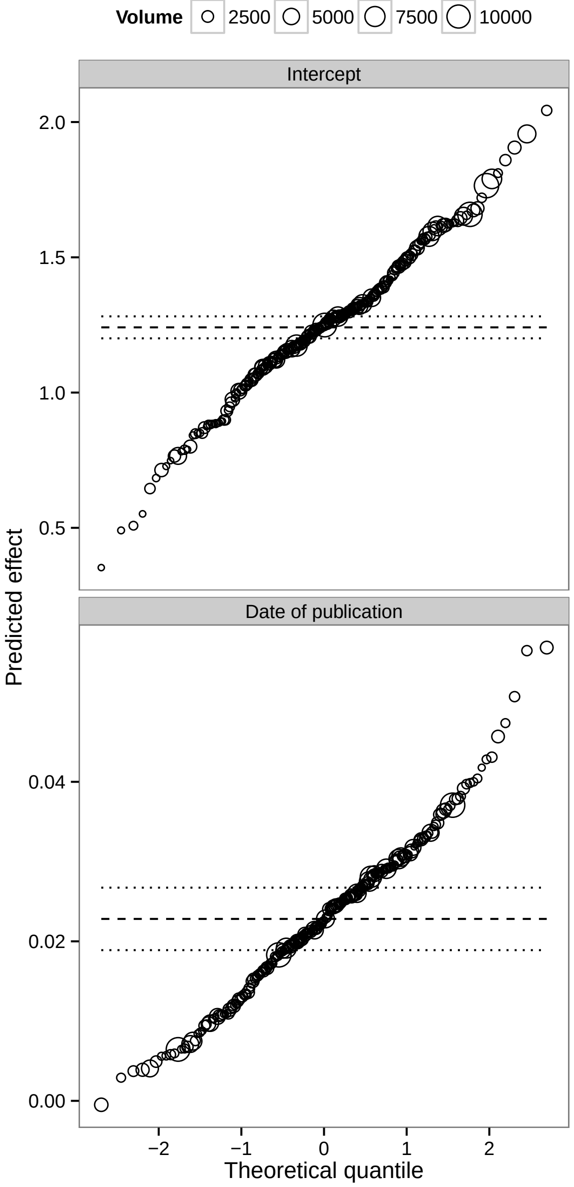

Figure 8: Normal QQ plot of journal-level effects (random effects added to fixed effect estimates) in the mixed model. The horizontal lines indicate the fixed estimate and its 99% confidence interval. Each circle's area is proportional to the corresponding journal's cumulative article count over the observation period. The journal with exceptionally high coauthor growth rate is the *Canadian Journal of Cardiology*, which jumped from an average 2.9 authors per article in 2002 to 4.3 in 2003, in contrast to steady behavior before and after .

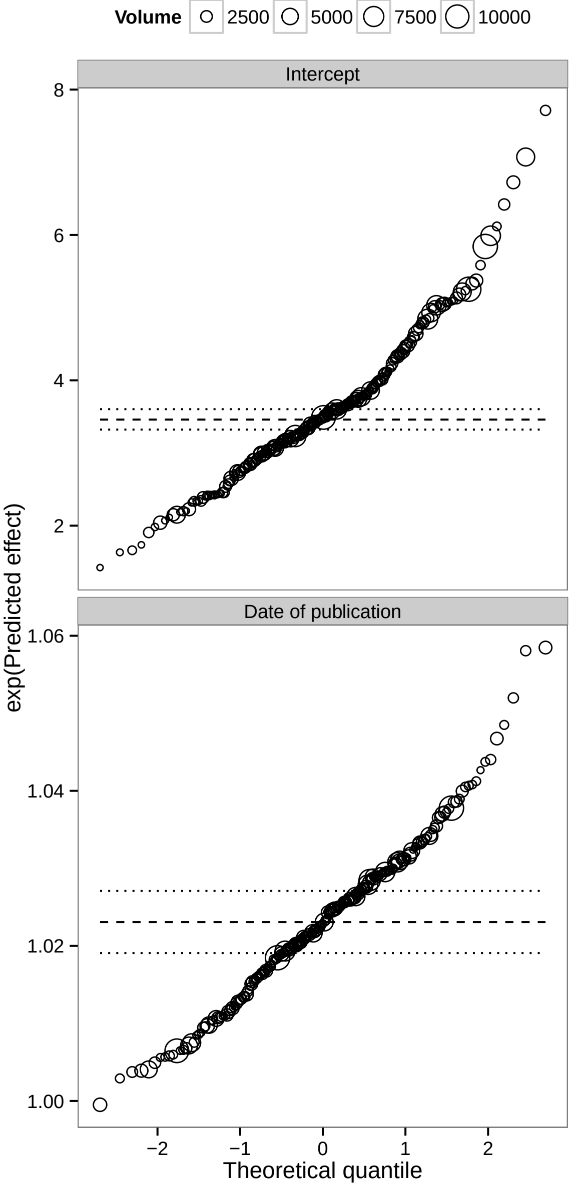

Figure 9: Normal QQ plot of exponentiated journal-level effects in the mixed model. Formatting is as in Figure 8.

Sensitivity analyses

The remainder of this supporting text summarizes the results of our several sensitivity analyses.

Lognormal models Citation count data have been found to follow lognormal distributions, and lognormal regression models on lognormal data have been shown to improve the accuracy of effect estimates over negative binomial models, in particular by reducing the false positive error rate. While it has not, to our knowledge, been carefully established that coauthor counts are well-modeled by a lognormal distribution, we fit a lognormal model to our data for the sake of comparison. Under proper assumptions, the lognormal model reduces the false positive error rate, so effects that are statistically weakened in this model are candidate (though not necessarily likely) false positives.

The results in Table 5 are highly suggestive: While the main effects are similar to those of the main analysis, in terms of discernibility, some of the interaction effects are no longer discernible (at the 99% threshold)—specifically, those we associated with research complexity, $\hat{\beta}_{DP\times CT}$ and $\hat{\beta}_{DP\times LMT}$.

Alternative variable measures As mentioned in the Methods section of the main text, we considered multiple measures for some variables and chose for the main analysis those that best satisfied the independence assumption of the model. To test the robustness of this choice, we fit the main models to the dataset obtained using each combination of measure choices. The measures are described in the main text and recalled in the caption of Fig 10.

Our results depended quantitatively, but not so much qualitatively, on our choice of measure for each variable. Figure 10 mimics Figure 4 in the main text, with the effect estimates obtained from the mixed-effects model using each combination of measures of funding, topical scope, and journal impact. While fluctuations are visible, the directions of the statistically discernible effects are the same in each fit, and their magnitudes are stable to within a factor of 2. Note that the estimated effect of grant acknowledgment on coauthorship growth (the alternative to $\hat{\beta}_{DP\times RS}$) was statistically obscured at the 99% confidence level. Ultimately, our choices of measures, with the partial exception of funding, did not affect our conclusions.

Removing corporate–(co)authored articles We included articles (co)written by corporate authors and consortia in the main analysis, though they were explicitly not our primary focus (which was the secular trend), yet they are often discussed as possible drivers of increasing authorship. Indeed, since PubMed started tagging corporate authors in 2001 (`https://www.nlm.nih.gov/bsd/disted/pubmedtutorial/020_770.html`), they have been credited by a steady 1.1% ± 0.1% of all indexed articles and averaged about 6 additional authors per article when counting each consortium "author" only once (as our analysis did). We use this flag to produce a dataset with corporate-(co)authored articles removed and reproduce the analysis. Unfortunately, we cannot discern from a corporate author credit how many individuals contributed to the study in a manner that might warrant coauthorship. Furthermore, while we verified manually that corporate authorship is highly correlated with consortium authorship, we identified both individual authors tagged as corporate and consortium authors not so tagged. Nevertheless, the effect on overall coauthorship rates from this subset of articles should give us a clear idea of whether the impact of consortium authorship is large and, if so, which direction it takes.

The results are tabulated in Table 6. It is clear that only minimal differences exist between these estimates and those of the main analysis.

11-year analysis using the impact factor Table 7 displays the effect estimates for the analysis over the observation window 1997–2007, during which journal impact factors were available but not SCImago journal rank indicators. The estimates reinforce the long-term trends in our competition-related factors (grant funding, impact factor) but call those in our complexity-related factors (study type, major MeSH term count) into question. In particular,

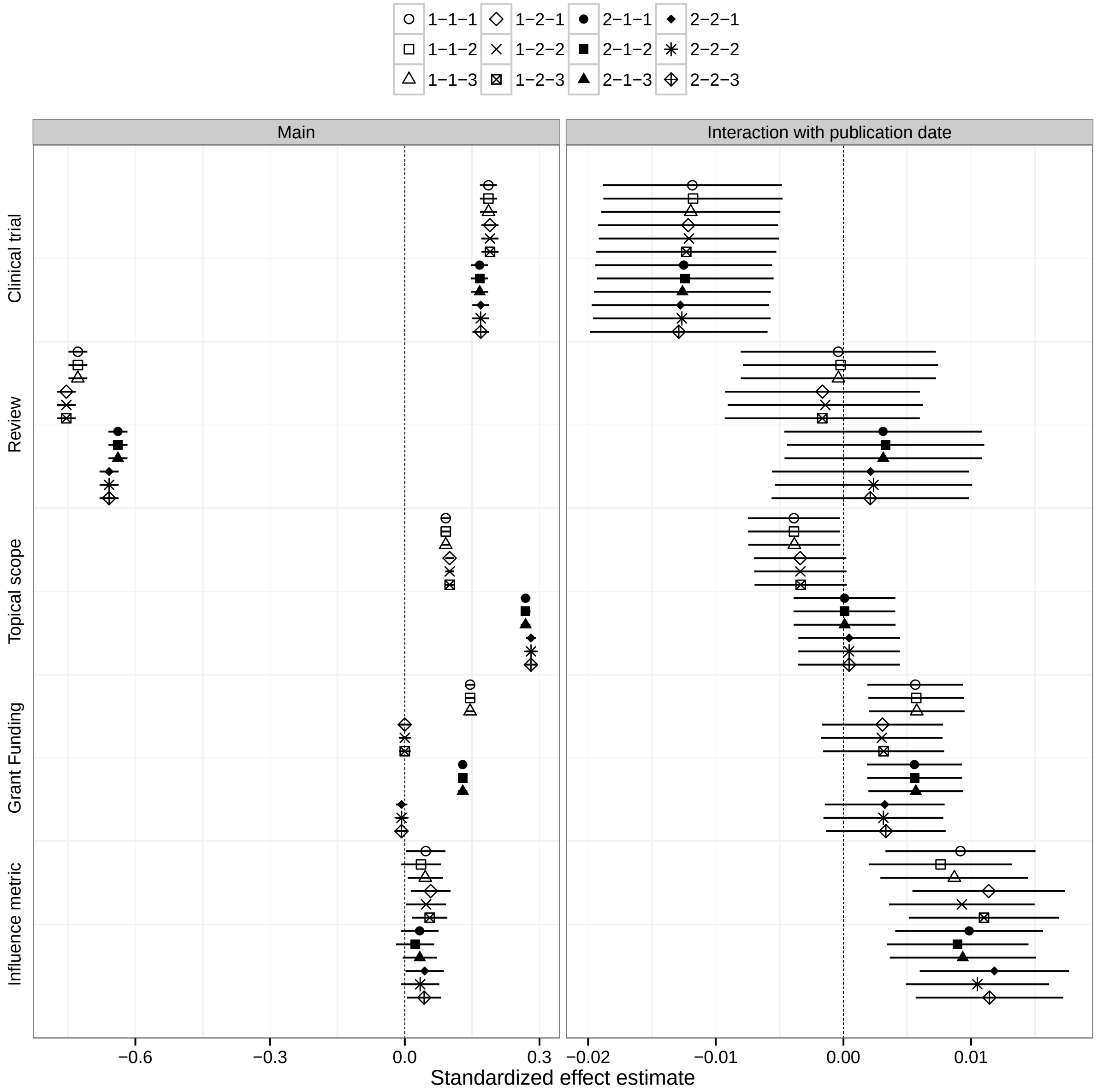

Figure 10: Comparison of standardized main and DP interaction effect estimates from the mixed model, with 99% confidence intervals, using each combination of measures. The combinations run over measures of topical scope (1 = number of major MeSH terms, 2 = number of top-level MeSH terms), research support (1 = research support Publication Type, 2 = acknowledgment of at least one grant), and citation metric (1 = impact factor, 2 = SCImago journalrank indicator, 3 = mean article two-year citation count). To compare the estimates for a single effect across all measures, look at a contiguous batch according to the labels on the left axis. To compare the estimates for all effects in a single model, look at the intervals around points plotted using the appropriate common symbol.

Predictor	Single confounder Main	Single confounder $DP\times$	Fixed effects Main	Fixed effects $DP\times$	Mixed effects Main	Mixed effects $DP\times$
Intercept (2003)	–	–	1.397 (.003)	.013 (.001)	1.348 (.012)	.016 (.001)
Clinical trial	1.430 (.036)	−.036 (.014)	.221 (.007)	−.006 (.003)	.167 (.007)	−.006 (.002)
Review	−2.441 (.028)	−.059 (.011)	−.629 (.005)	−.001 (.002)	−.587 (.005)	−.003 (.002)
log(Major MeSH + 1)	1.130 (.023)	−.031 (.009)	.173 (.004)	−.013 (.002)	.138 (.004)	−.005 (.002)
Support	.829 (.017)	.043 (.006)	.143 (.003)	.006 (.001)	.163 (.003)	.006 (.001)
log(Impact factor)	.565 (.011)	.056 (.004)	.057 (.002)	.003 (.001)	.019 (.009)	.002 (.001)

Table 5: Effect estimates, with standard errors, for lognormal alternatives to each model fitted to main dataset.

Predictor	Single confounder Main	Single confounder $DP\times$	Fixed effects Main	Fixed effects $DP\times$	Mixed effects Main	Mixed effects $DP\times$
Intercept (2003)	–	–	1.345 (.003)	.019 (.001)	1.237 (.016)	.024 (.001)
Clinical trial	.288 (.008)	−.013 (.002)	.179 (.005)	−.015 (.002)	.177 (.007)	−.010 (.003)
Review	−.866 (.008)	.000 (.003)	−.807 (.008)	−.003 (.003)	−.738 (.008)	−.006 (.003)
log(Major MeSH + 1)	.301 (.006)	−.013 (.002)	.179 (.005)	−.015 (.002)	.135 (.005)	−.004 (.002)
Support	.205 (.004)	.005 (.001)	.100 (.004)	.005 (.001)	.140 (.004)	.006 (.001)
log(Impact factor)	.130 (.003)	.010 (.001)	.108 (.003)	.009 (.001)	.023 (.011)	.006 (.001)

Table 6: Effect estimates, with standard errors, for each model fitted to data with articles (co)written by corporate authors removed.

note that the slightly longer view renders the DP-interaction effect of LMT indiscernible in the mixed model.

Impact factor as a journal-level term Though the impact factor is a journal-level variable, we model it as an article-level predictor, a choice that one reviewer reasonably questioned. An alternative is to include additional journal-level random effects, alongside publication date, as follows:

$$
\begin{aligned}
\log(E[NC_i]) = \beta_1 &+ \beta_{DP} DP_i \\
&+ (\beta_{RS} + \beta_{DP\times RS} DP_i) RS_i \\
&+ (\beta_{CT} + \beta_{DP\times CT} DP_i) CT_i \\
&+ (\beta_{RV} + \beta_{DP\times RV} DP_i) RV_i \\
&+ (\beta_{LMT} + \beta_{DP\times LMT} DP_i) LMT_i \\
&+ (\beta_{LIF} + \beta_{DP\times LIF} DP_i) LIF_{j[i]} \\
&+ (b_{1j[i]} + b_{DP,j[i]} DP_i + b_{LIF,j[i]} LIF_i)
\end{aligned}
$$

As with the other two terms, we take $B_{LIF,j} \sim N(0, {\sigma_{LIF}}^2)$.

Table 8 displays the effect estimates for the two main models and for the model above (the single-confounder comparisons are omitted). The main and DP-interaction effect estimates, including $\hat{\beta}_{LIF}$, are minimally changed, including those of $LIF_{j[i]}$. Table 9 displays the estimated standard deviations of these two models. We estimated $\hat{\sigma}_{LIF} = .11$, compared to $\hat{\sigma}_1 = .25$ and $\hat{\sigma}_{DP} = .013$, indicating a strong effect of impact factor on how journals are ranked by coauthorship, and independently from their article-level effect. Again, the inclusion of $B_{LIF,j}$ had little effect on the roles of the other random effects. Overall, this suggests that citation metrics measure distinct journal-level and article-level influences on coauthorship rates.

Rank index indicator The restriction to journals indexed by WoS (and SCImago) greatly reduces our sample and biases it toward journals that meet the indexing criteria and apply to be indexed, which are likely to be both more rigorous and more popular. We duced the main analysis with the additional predictor variable $CC_{j[i]}$, an indicator for whether article i was published in a journal $j = j[i]$ included

Predictor	Single confounder Main	×DP	Fixed effects Main	×DP	Mixed effects Main	×DP
Intercept (2003)	–	–	1.352 (.0013)	.014 (.0004)	1.261 (.0110)	.020 (.0007)
Clinical trial	.282 (.0032)	−.001 (.0010)	.238 (.0030)	−.003 (.0010)	.183 (.0030)	−.004 (.0009)
Review	−.828 (.0035)	.002 (.0011)	−.749 (.0035)	−.002 (.0011)	−.705 (.0035)	−.001 (.0011)
log(Major MeSH + 1)	.281 (.0022)	−.03 (.0007)	.164 (.0022)	−.004 (.0007)	.132 (.0022)	.000 (.0007)
Support	.193 (.0016)	.009 (.0005)	.075 (.0017)	.008 (.0005)	.135 (.0017)	.008 (.0005)
log(Impact factor)	.169 (.0011)	.008 (.0004)	.134 (.0012)	.007 (.0004)	.046 (.0061)	.005 (.0007)

Table 7: Effect estimates, with standard errors, for each model fitted to data over 1997–2007.

Predictor	Fixed effects Main	×DP	Mixed effects Main	×DP	Mixed effects (w/ LIF) Main	×DP
Intercept (2003)	1.349 (.003)	.018 (.001)	1.241 (.016)	.023 (.002)	1.243 (.0157)	.023 (.002)
Clinical trial	.251 (.008)	−.013 (.003)	.186 (.007)	−.012 (.003)	.186 (.007)	−.012 (.003)
Review	−.793 (.008)	.001 (.003)	−.728 (.008)	−.000 (.003)	−.728 (.008)	−.000 (.003)
log(Major MeSH + 1)	.120 (.004)	−.011 (.003)	.091 (.004)	−.004 (.003)	.091 (.004)	−.004 (.001)
Support	.104 (.004)	.004 (.002)	.146 (.004)	.006 (.001)	.145 (.004)	.006 (.001)
log(Impact factor)	.164 (.004)	.014 (.001)	.047 (.017)	.009 (.002)	.048 (.019)	.009 (.002)

Table 8: Effect estimates, with standard errors, for the main analysis models together with the model including LIF as a random effect.

in the WoS Core Collection (`http://wokinfo.com/essays/journal-selection-process/`), along with its own DP-interaction term, and we set $LIF_{j[i]} = 0$ when $CC_{j[i]} = 0$. This addresses a limitation of the main analysis, for which the data were necessarily restricted to journals included in the Core Collection, namely that the effect of impact factor *within* the Core Collection may be smaller than the difference in coauthorship rates *between* the Core Collection and those biomedical journals not indexed there.

Estimates of other effects were roughly consistent with the main analysis; the exception was a discernibly positive interaction effect $\hat{\beta}_{DP\times RV}$, suggesting that the main effect of study type weakened over time (since $\hat{\beta}_{RV} < 0$). We estimated a strong effect of inclusion $\hat{\beta}_{CC} = .733 \pm .038$ in the mixed model, exceeding even $\hat{\beta}_{RV} = -.613$. We estimated a zero interaction effect $\hat{\beta}_{DP\times CC} = .000 \pm .0018$ in tandem with a positive (de-standardized) journal popularity interaction effect $\hat{\beta}_{DP\times IF} = .002 \pm .0008$. However, the model was a poor fit to the data, so we take these numbers cautiously.

	Mixed effects	Mixed effects (w/ LIF)
Intercept (2003)	.2588	.2504
Publication date	.0130	.0129
log(Impact factor)	–	.1149

Table 9: Estimated standard deviations of the random effects in two mixed-effects models..
