# Supplementary material for: Effects of research complexity and competition on the incidence and growth of coauthorship in biomedicine
Source: PLoS One. 2017 Mar 22;12(3):e0173444. doi: 10.1371/journal.pone.0173444 (PMC5362051; doi:10.1371/journal.pone.0173444)
Supplement: S1 Fig — The SCs are listed first in order of the number of journals in our dataset to which they were assigned, then alphabetically. See S1 Text for the number of journals in each SC. See S1 Text for the number of journals in each SC. (PDF) [file pone.0173444.s002.pdf]

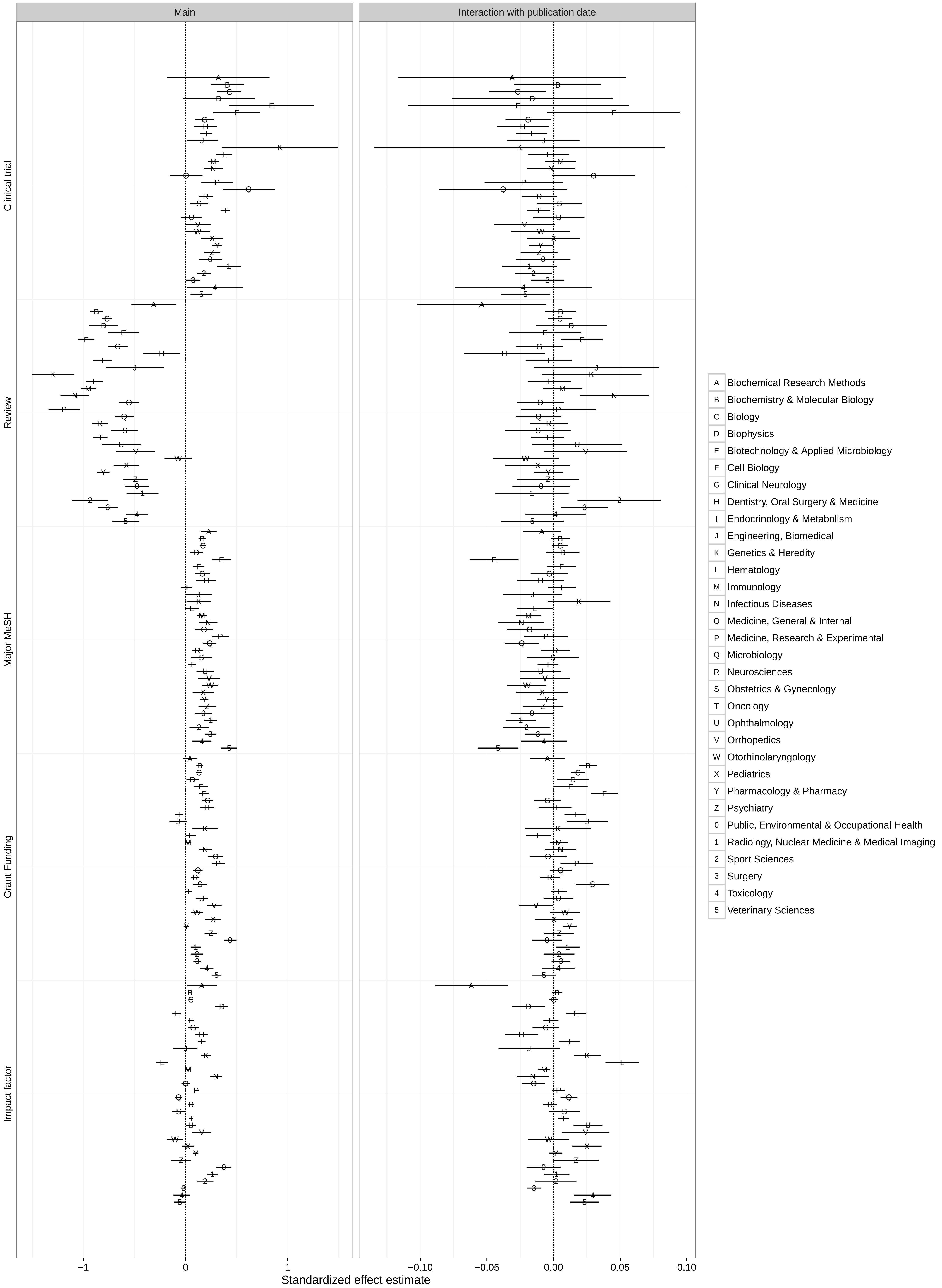

Main
Interaction with publication date
Clinical trial
Review
Major MeSH
Grant Funding
Impact factor
−1
0
1
−0.10
−0.05
0.00
0.05
0.10
Standardized effect estimate
A Biochemical Research Methods
B Biochemistry & Molecular Biology
C Biology
D Biophysics
E Biotechnology & Applied Microbiology
F Cell Biology
G Clinical Neurology
H Dentistry, Oral Surgery & Medicine
I Endocrinology & Metabolism
J Engineering, Biomedical
K Genetics & Heredity
L Hematology
M Immunology
N Infectious Diseases
O Medicine, General & Internal
P Medicine, Research & Experimental
Q Microbiology
R Neurosciences
S Obstetrics & Gynecology
T Oncology
U Ophthalmology
V Orthopedics
W Otorhinolaryngology
X Pediatrics
Y Pharmacology & Pharmacy
Z Psychiatry
0 Public, Environmental & Occupational Health
1 Radiology, Nuclear Medicine & Medical Imaging
2 Sport Sciences
3 Surgery
4 Toxicology
5 Veterinary Sciences
