# Supplementary figures and images for: Effects of research complexity and competition on the incidence and growth of coauthorship in biomedicine
Source: PLoS One. 2017 Mar 22;12(3):e0173444. doi: 10.1371/journal.pone.0173444 (PMC5362051; doi:10.1371/journal.pone.0173444)

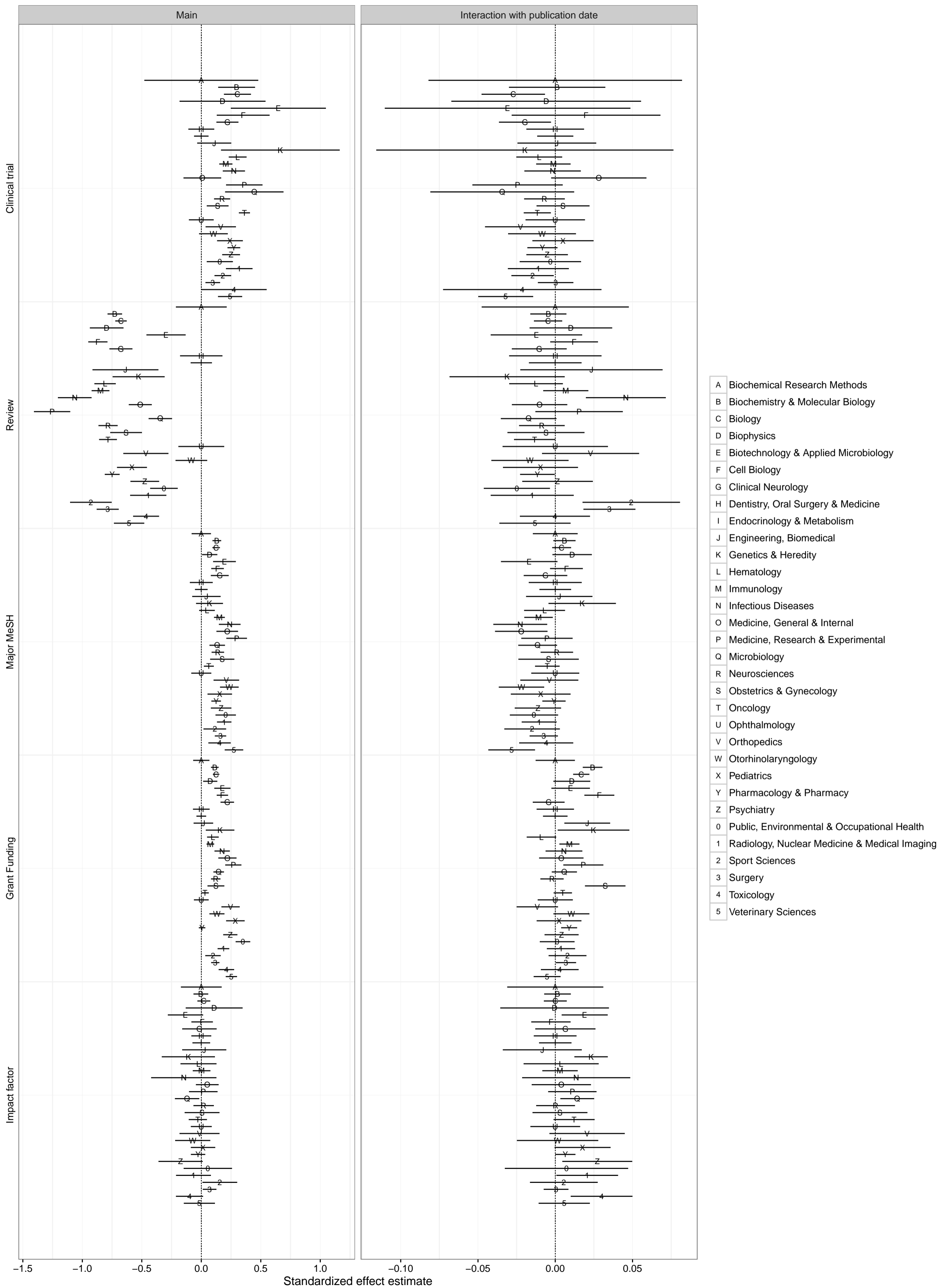

Supplement: S2 Fig — The SCs are listed first in order of the number of journals in our dataset to which they were assigned, then alphabetically. See S1 Text for the number of journals in each SC. (PDF) [file pone.0173444.s003.pdf]
